# Autophagy stimulation reduces ocular hypertension in a murine glaucoma model via autophagic degradation of mutant myocilin

**DOI:** 10.1172/jci.insight.143359

**Published:** 2021-03-08

**Authors:** Ramesh B. Kasetti, Prabhavathi Maddineni, Charles Kiehlbauch, Shruti Patil, Charles C. Searby, Beth Levine, Val C. Sheffield, Gulab S. Zode

**Affiliations:** 1 Department of Pharmacology and Neuroscience and the North Texas Eye Research Institute, University of North Texas Health Science Center at Fort Worth, Fort Worth, Texas, USA.; 2 Department of Pediatrics, Carver College of Medicine, University of Iowa, Iowa City, Iowa, USA.; 3 Center for Autophagy Research, Department of Internal Medicine,; 4 Howard Hughes Medical Institute, and; 5 Department of Microbiology, University of Texas Southwestern Medical Center, Dallas, Texas, USA.

**Keywords:** Cell Biology, Ophthalmology, Autophagy, Cell stress, Protein misfolding

## Abstract

Elevation of intraocular pressure (IOP) due to trabecular meshwork (TM) damage is associated with primary open-angle glaucoma (POAG). Myocilin mutations resulting in elevated IOP are the most common genetic causes of POAG. We have previously shown that mutant myocilin accumulates in the ER and induces chronic ER stress, leading to TM damage and IOP elevation. However, it is not understood how chronic ER stress leads to TM dysfunction and loss. Here, we report that mutant myocilin activated autophagy but was functionally impaired in cultured human TM cells and in a mouse model of myocilin-associated POAG (*Tg-MYOC^Y437H^*). Genetic and pharmacological inhibition of autophagy worsened mutant myocilin accumulation and exacerbated IOP elevation in *Tg-MYOC^Y437H^* mice. Remarkably, impaired autophagy was associated with chronic ER stress–induced transcriptional factor CHOP. Deletion of CHOP corrected impaired autophagy, enhanced recognition and degradation of mutant myocilin by autophagy, and reduced glaucoma in *Tg-MYOC^Y437H^* mice. Stimulating autophagic flux via tat-beclin 1 peptide or torin 2 promoted autophagic degradation of mutant myocilin and reduced elevated IOP in *Tg-MYOC^Y437H^* mice. Our study provides an alternate treatment strategy for myocilin-associated POAG by correcting impaired autophagy in the TM.

## Graphical abstract

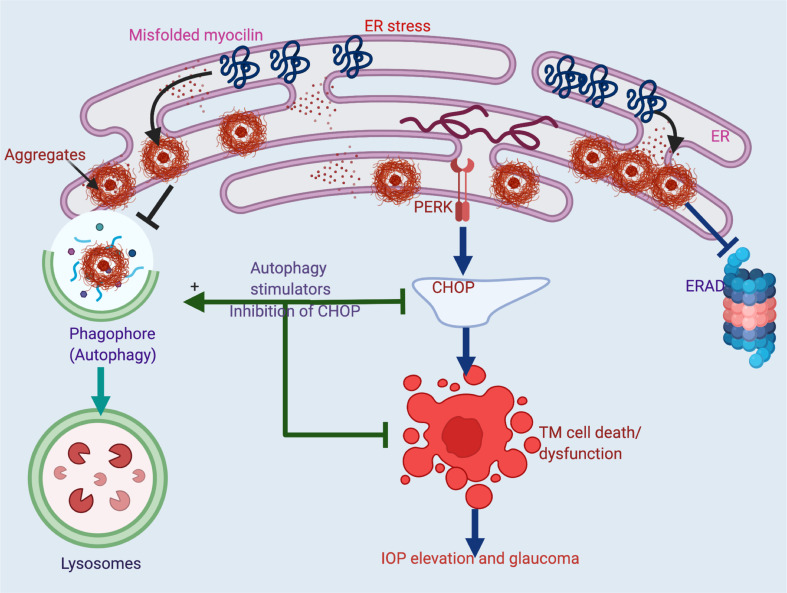

## Introduction

Glaucoma is characterized by the progressive loss of retinal ganglion cells, degeneration of the optic nerve, and progressive visual field loss (1–3). Primary open-angle glaucoma (POAG) is the most common form of glaucoma and is especially prevalent in African American and Hispanic populations (4–8). Elevated intraocular pressure (IOP) is a major associated risk factor, and currently the only treatable risk factor (9). The trabecular meshwork (TM) maintains normal IOP by regulating outflow resistance. In POAG, there is increased resistance to aqueous humor outflow through the TM, thus elevating IOP (2, 10). This increase in outflow resistance in glaucoma is associated with dysfunction and loss of TM cells (2, 11). However, the exact pathological mechanisms that lead to glaucomatous TM damage are not fully understood.

Myocilin mutations are responsible for approximately 4% of POAG and most cases of autosomal dominant juvenile-onset open-angle glaucoma (2, 12, 13). The exact role of WT myocilin is not completely understood. Various genetic strategies, including knockout, knockin, and expression of transgenic mutant myocilin, have been used to generate mouse models (14–20). These studies demonstrated that WT myocilin is not required for the physiological regulation of IOP and suggested that myocilin-associated glaucoma is the result of a gain of function or novel property of mutant myocilin (16, 21, 22). We generated a transgenic mouse model (*Tg-MYOC^Y437H^*) by expressing human myocilin containing the Y437H mutation under the control of the CMV promoter (23). *Tg-MYOC^Y437H^* mice express myocilin in the TM and display early-onset glaucoma phenotypes (23). We and others have shown that disease-causing myocilin mutant proteins are secretion incompetent and accumulate in the ER, inducing ER stress (16, 21–28). Chronic ER stress is known to play a key role in the elevation of IOP in myocilin-associated glaucoma (23). Specifically, induction of chronic ER stress–induced transcription factor, CCAAT/enhancer-binding protein homologous protein (CHOP), is associated with a loss of TM cells (23). However, it is not understood how CHOP leads to TM loss/dysfunction and IOP elevation.

When the levels of misfolded proteins overwhelm normal protein degradation pathways, cells can activate autophagy, a lysosomal-mediated degradation pathway. Several studies have revealed interaction between the ER stress response pathway and autophagy (29–31). Autophagy is responsible for the removal of protein aggregates, long-lived proteins, and defective organelles (32–34). During the autophagic process, cargos are engulfed within a double membrane vesicle called an autophagosome. These autophagosomes fuse with lysosomes, and cargos are degraded via lysosomal enzymes. Beclin 1, a protein coded by the *BECN1* gene, forms a complex with phosphoinositide 3-kinase (PI3K), which is required for the initiation of autophagosome formation. Microtubule-associated protein light chain 3 (LC3) is a major regulator of autophagosome formation, and conversion of LC3B-I to LC3B-II is an indicator of autophagosome formation (35). In addition, the ubiquitin binding protein p62, also known as sequestosome 1 (SQSTM1), binds directly to LC3B, which is then degraded by autophagy and may serve as a marker to study autophagic degradation and autophagic flux. Autophagy plays an important role in preventing toxic accumulation of disease-associated mutant proteins (28, 36–38). Several previous studies have shown that ER stress induces autophagic response and ER stress–induced autophagy is cytoprotective (29, 30, 39–41). Despite activation of the protective unfolded protein response (UPR) pathway and autophagy, misfolded proteins continue to accumulate in disease conditions. It is not yet understood whether chronic and persistent ER stress can lead to impaired autophagy.

A previous study has shown involvement of both ubiquitin-proteasome and lysosomal pathways in turnover of endogenous WT myocilin in cultured TM cells (42). However, proteosomal degradation of mutant myocilin is impaired and autophagy is activated. It is not understood why autophagy fails to degrade mutant myocilin and whether autophagy plays a critical role in mutant myocilin–induced ocular hypertension. We have recently shown that induction of chronic ER stress leads to glaucoma by increasing protein synthesis and ER client protein load in TM cells (43). These events can further lead to compromised autophagy. We propose that induction of CHOP is associated with compromised autophagy, leading to mutant myocilin accumulation in TM cells, causing TM dysfunction/loss and IOP elevation in a mouse model of myocilin POAG. Here, we investigated whether mutant myocilin–induced chronic ER stress leads to impaired autophagy and whether correction of autophagic flux via deletion of CHOP or pharmacological autophagy inducers promotes autophagic degradation of mutant myocilin and reduces elevated IOP in a mouse model of myocilin-associated glaucoma.

## Results

### Mutant myocilin–induced chronic ER stress is associated with impaired autophagy in TM cells in vitro and in vivo.

**Figure 1:**
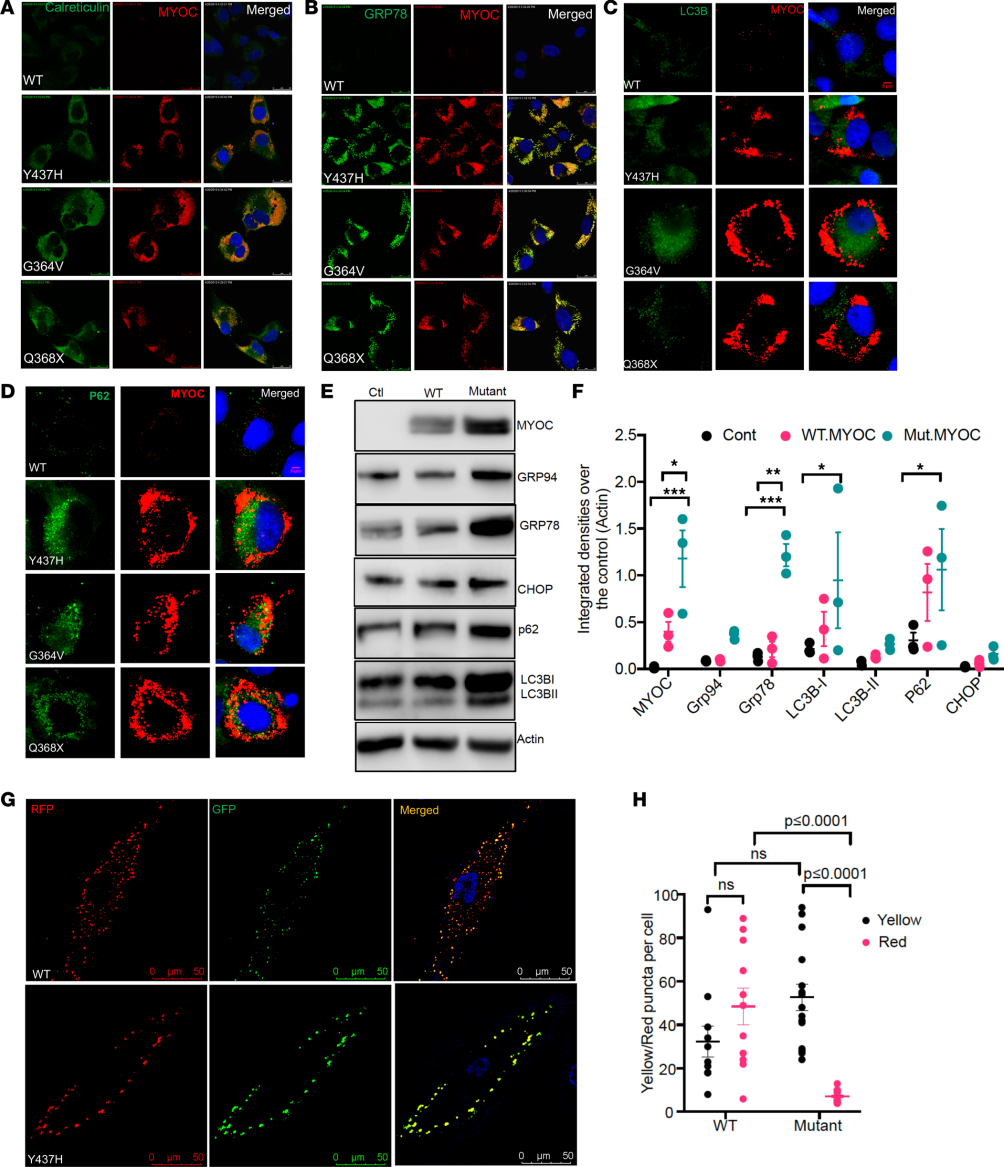
Mutant myocilin leads to impaired autophagy, which is associated with chronic ER stress in human cultured TM cells. (**A** and **B**) TM3 cells stably expressing DsRed-tagged WT or individual myocilin mutations (Y437H, G364V, and Q368X) were stained with ER marker, calreticulin (**A**), or ER stress marker GRP78 (**B**). All mutants of myocilin colocalized strongly with calreticulin and GRP78, indicating that mutant myocilin accumulated in the ER and induced ER stress. *n* = 3. Scale bar: 5 μm. (**C** and **D**) TM3 cells stably expressing WT or Y437H, G364V, and Q368X mutants of myocilin were stained with LC3B (**C**) and p62 (**D**). Compared with WT myocilin, TM cells stably expressing mutant myocilin exhibit increased LC3B and p62 staining. *n* = 3. Scale bar: 5 μm. (**E** and **F**) Primary human TM cells were transduced with Ad5 expressing empty (ctl), WT, or Y437H mutant myocilin. Western blot (**E**) and densitometric analysis (**F**) were performed to determine autophagy and ER stress. Expression of mutant myocilin resulted in impaired autophagy and chronic ER stress, as evident from increased intracellular myocilin, p62, LC3BI, GRP78, and CHOP in TM cells transduced with mutant myocilin. *n* = 3 cell strains; data are mean ± SEM; * *P* ≤ 0.05; ** *P* ≤ 0.01; ***P ≤ 0.01; 2-way ANOVA. (**G** and **H**) Primary human TM cells were transduced with Ad5 expressing WT or Y437H mutant myocilin for 48 hours and again transduced with Ad5.mRFP-GFP-LC3 for 24 hours. Autophagosomes (yellow puncta) and autolysosomes (red puncta) were counted. Expression of mutant myocilin resulted in impaired autophagic flux as evident from increased yellow puncta and significantly reduced red puncta compared with WT myocilin. *n* = 3 cell strains; data are mean ± SEM; 2-way ANOVA; Scale bar: 50 μm.

To study the effect of mutant myocilin on chronic ER stress and autophagy, we generated TM3 cells stably expressing DsRed-tagged WT or various individual myocilin mutations (Y437H, G364V, and Q368X). Y437H and G364V myocilin mutations represent more severe and early onset POAG, whereas Q368X represents less severe and later onset POAG (44). Consistent with our previous studies (45, 46), all mutants of myocilin accumulated in the ER, as evident from colocalization of mutant myocilin with calreticulin (Figure 1A) and KDEL antibodies (Supplemental Figure 1A; supplemental material available online with this article; https://doi.org/10.1172/jci.insight.143359DS1). Expression of mutant myocilin induced chronic ER stress and TM cell death, as evident from increased GRP78 (Figure 1B), CHOP, and cleaved PARP (Supplemental Figure 1B). There was strong colocalization of mutant myocilin with GRP78, calreticulin, and KDEL, indicating the presence of misfolded protein. We also observed that expression of various mutants of myocilin impaired autophagy, as evident from increased p62 and LC3B, which was associated with induction of CHOP (Figure 1, C and D, and Supplemental Figure 2). Although LC3B puncta were increased in TM3 cells expressing mutant myocilin, indicating activation of autophagy, there was little or no colocalization of LC3B with myocilin and p62 levels were dramatically increased. We further explored mutant myocilin–induced impaired autophagy using primary human TM cells (*n* = 3 donor strains). Human primary TM cells were transduced with adenovirus 5 (Ad5) expressing empty, WT, or Y437H mutant human myocilin. Western blot and densitometric analysis (Figure 1, E and F) demonstrated significantly increased myocilin, GRP78, p62, and LC3B in primary TM cells expressing mutant (Y437H) myocilin compared with WT myocilin, indicating that expression of mutant myocilin led to activation of autophagy, but impaired autophagy progression. The presence of mutant myocilin also appeared to increase GRP94 and CHOP. However, the degree of increase of these markers was not statistically significant, likely because of large variations in 3 cell strains from different donor eyes.

Tandem fluorescently tagged LC3 (RFP-GFP-LC3) is used widely to monitor autophagic flux based on the stability of GFP and RFP fluorescent proteins at different pH levels. Primary human TM cells (*n* = 3 donor strains) were transduced with Ad5 expressing either WT or Y437H mutant myocilin for 48 hours and transduced again with Ad5 expressing RFP-GFP-LC3 for 24 hours. RFP and GFP were examined using confocal microscopy and LC3 puncta were quantified (Figure 1, G and H). TM cells expressing mutant myocilin demonstrated significantly increased yellow puncta (autophagosomes) and reduced red puncta (autolysosomes), indicating reduced autophagic flux and accumulation of autophagosomes. Compared with TM cells expressing WT myocilin, TM cells expressing mutant myocilin exhibited a significantly reduced number of autolysosomes. Together, these data clearly indicate that expression of mutant myocilin activated autophagy, but autophagy function was impaired. The impairment of autophagy was associated with chronic ER stress in cultured TM cells.

**Figure 2:**
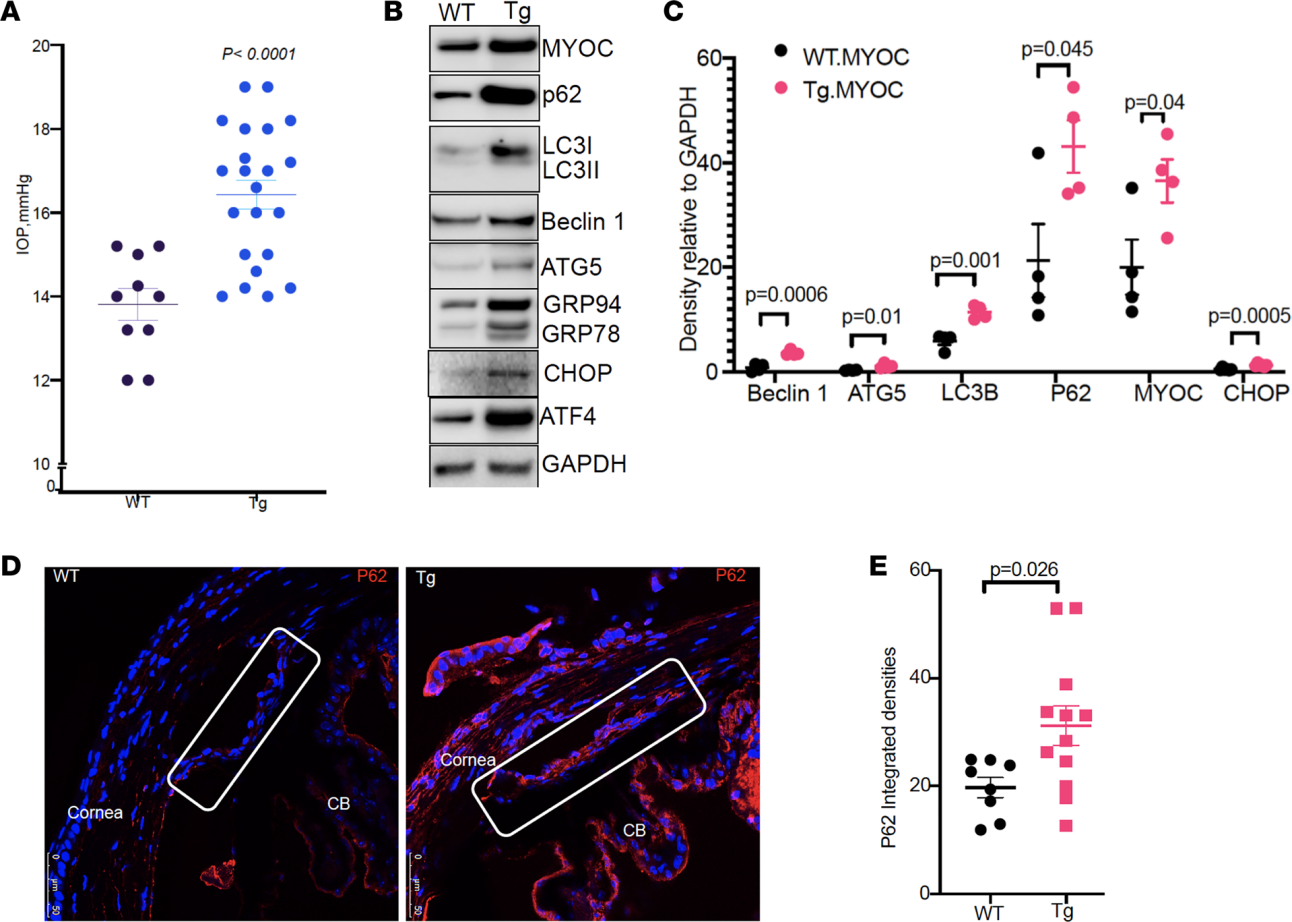
Impaired autophagy is associated with chronic ER stress in TM tissues of *Tg-MYOC^Y437H^* mice. (**A**) Conscious IOP measurements of WT and *Tg-MYOC^Y437H^* littermates demonstrated that 4-month-old *Tg-MYOC^Y437H^* mice developed significant ocular hypertension. Data are mean ± SEM; *n* = 10 in WT and *n* = 22 in *Tg-MYOC^Y437H^* mice; unpaired *t* test. (**B** and **C**) Western blot (**B**) and densitometric analyses (**C**) of anterior segment tissue lysates from 4-month-old WT and *Tg-MYOC^Y437H^* mice demonstrated activated autophagy but autophagy function was impaired (significantly increased LC3BI and p62), which was associated with induction of chronic ER stress, as evident from significantly increased ATF4 and CHOP. *n* = 4 mice for myocilin, LC3B, p62, Beclin1, ATG5 and *n* = 6 for CHOP; data are mean ± SEM; unpaired *t* test. (**D** and **E**) Immunostaining and intensity measurements for p62 in anterior segment tissues of WT and *Tg-MYOC^Y437H^* mice demonstrated significantly increased p62 in mouse TM tissues of *Tg-MYOC^Y437H^* mice. Scale bar: 50 μm. Rectangular box indicates TM. CB, ciliary body. Data are mean ± SEM; *n* = 8 in WT and *n* = 12 in *Tg-MYOC^Y437H^* mice; *P =* 0.026; unpaired *t* test.

Next, we examined whether mutant myocilin–induced impaired autophagy in the TM is associated with ocular hypertension in *Tg-MYOC^Y437H^* mice. Conscious IOP measurements demonstrated that 4-month-old *Tg-MYOC^Y437H^* mice developed significant ocular hypertension (Figure 2A). Western blot and densitometric analysis of anterior segment tissues revealed significant increases in myocilin and chronic ER stress markers, as evident from increased GRP78, GRP94, and CHOP in *Tg-MYOC^Y437H^* mice compared with WT littermates (Figure 2, B and C). *Tg-MYOC^Y437H^* mice also demonstrated significantly increased ATG5 and beclin1, indicating activation of autophagy. Similar to cultured TM cells, we observed a significant increase in p62 and LC3BI in mouse anterior segment tissue, further indicating impaired autophagic flux in *Tg-MYOC^Y437H^* mice. We next examined p62 levels in TM tissues of WT and *Tg-MYOC^Y437H^* mice (Figure 2D). Immunostaining and analysis (Figure 2, D and E) of p62 revealed significantly increased p62 in anterior segment tissues of *Tg-MYOC^Y437H^* mice compared with WT littermates. These data indicate that expression of mutant myocilin led to impaired autophagy in TM cells in vitro and in vivo.

### Inhibition of autophagy exacerbates mutant myocilin accumulation in TM cells and worsens IOP elevation in Tg-MYOC^Y437H^ mice.

**Figure 3:**
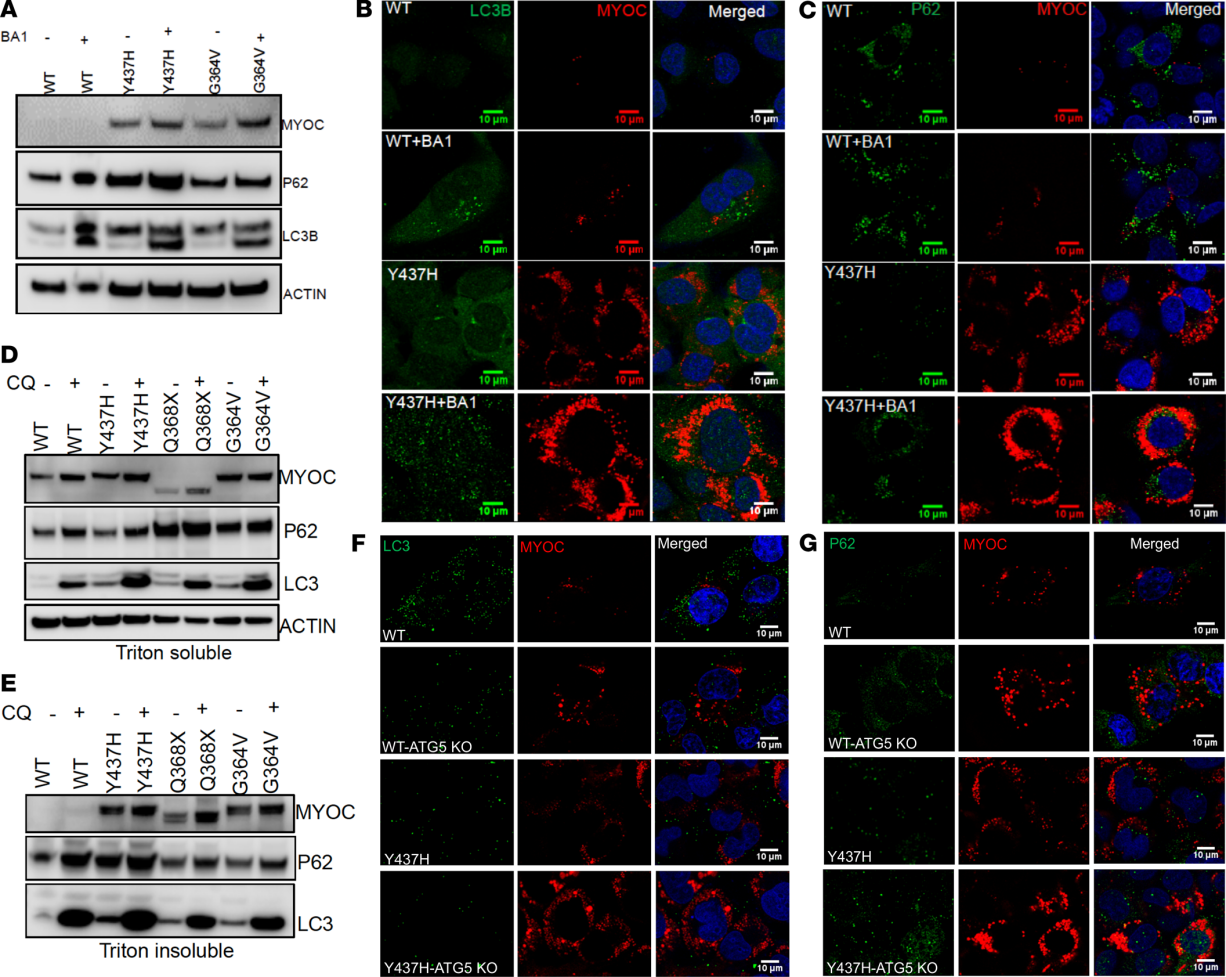
Inhibition of autophagy exacerbates WT and mutant myocilin accumulation in cultured TM cells. (**A**) TM3 cells stably expressing WT or mutant myocilin (Y437H or G364V) were treated with BA1 (200 nM) for 6 hours and cellular lysates were examined for intracellular myocilin accumulation and autophagy markers. Treatment with BA1 inhibited autophagic degradation of both WT and mutant myocilin in TM3 cells. *n* = 3. (**B** and **C**) TM3 cells stably expressing WT or mutant myocilin were treated with BA1 for 6 hours and cells were stained with LC3B (**B**) and p62 (**C**). BA1 treatment resulted in increased myocilin (WT and mutant), LC3B puncta, and P62 levels. *n* = 3. Scale bar: 10 μm. (**D** and **E**) TM3 cells stably expressing WT or various mutants of myocilin were treated with CQ (50 μM) for 12 hours. Triton X-100–soluble and –insoluble fractions were examined for intracellular myocilin accumulation and autophagy markers. CQ treatment inhibited autophagic degradation of mutant myocilin and increased accumulation of Triton X-100–soluble and –insoluble mutant myocilin. *n* = 3. (**F** and **G**) TM3 cells stably expressing WT or Y437H mutant myocilin were transfected with CRISPR/Cas9 targeting ATG5 and stained with LC3B and P62. ATG5 knockdown prevented autophagy induction and increased WT and mutant myocilin accumulation. *n* = 3. Scale bar: 10 μm.

We explored whether pharmacological or genetic inhibition of autophagy leads to increased mutant myocilin accumulation in cultured TM cells. Bafilomycin A1 (BA1) and chloroquine (CQ) are commonly used compounds that inhibit autophagy by targeting lysosomes (35, 47). TM3 cells stably expressing WT or various mutants of myocilin were treated with BA1(200 nM) for 6 hours and autophagy markers were analyzed by Western blot (Figure 3A) and immunostaining (Figure 3, B and C). Accumulation of autophagosomes in BA1-treated TM cells was evident from increased LC3B puncta and p62 accumulation. Inhibition of autophagy resulted in increased WT and mutant myocilin, suggesting that autophagy was involved in WT or mutant myocilin degradation. We further confirmed these results using CQ treatment. TM3 cells stably expressing WT or various mutants of myocilin were treated with CQ (50 μM) for 12 hours. Western blot analysis (Figure 3, D and E) and immunostaining (Supplemental Figure 3) demonstrated that CQ treatment increased intracellular accumulation of WT and mutant myocilin (Triton X-100 soluble and insoluble; Figure 3, D and E). Increased LC3B and p62 confirmed autophagosome accumulation after CQ treatment. Inhibition of autophagy via CQ treatment further increased intracellular accumulation of WT and the Y437H mutant of myocilin.

We next examined whether ATG5 knockdown exacerbates intracellular mutant myocilin accumulation in TM3 cells. TM3 cells stably expressing WT or mutant myocilin were transfected with CRISPR/Cas9 targeting ATG5 for 48 hours. Myocilin, LC3B, and p62 levels were determined by Western blot analysis (Supplemental Figure 4) and immunostaining (Figure 3, F and G). Western blot analysis confirmed that CRISPR/Cas9 targeting of ATG5 reduced ATG5 protein levels in TM3 cells expressing WT or mutant myocilin (Supplemental Figure 4, A and B). ATG5 knockdown resulted in prominent increases in intracellular mutant myocilin accumulation along with an increase in p62 levels in TM3 cells stably expressing WT or mutant myocilin (Figure 3, F and G). These data indicate that inhibition of autophagy exacerbated intracellular WT and mutant myocilin accumulation in TM cells.

**Figure 4:**
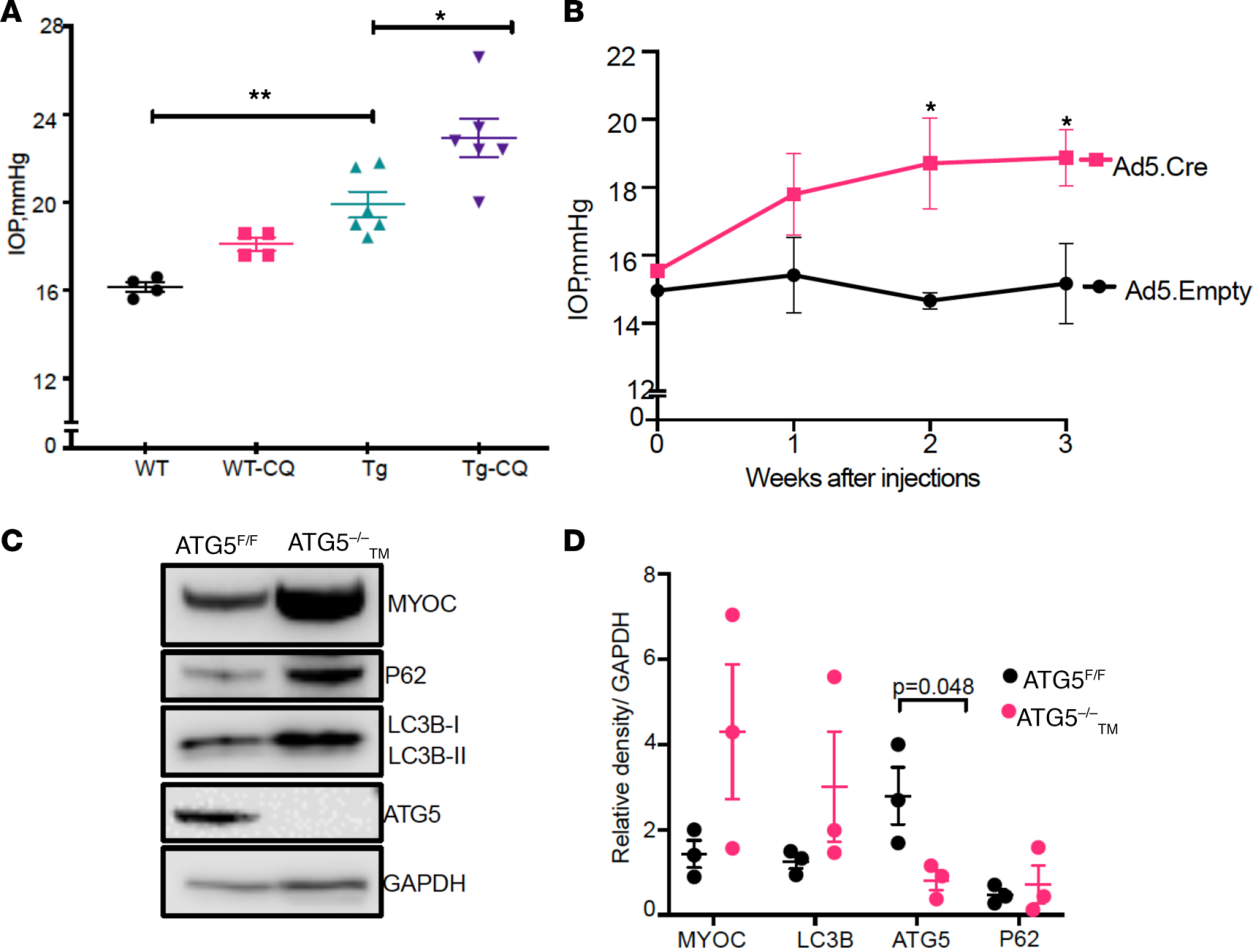
Inhibition of autophagy exacerbates mutant myocilin–induced IOP elevation in *Tg-MYOC^Y437H^* mice. (**A**) WT and *Tg-MYOC^Y437H^* littermates (4 months old) were given topical ocular vehicle in 1 eye and the contralateral eyes were treated with CQ (10 mM) eye drops for 7 days and IOP was monitored. CQ treatment elevated IOP in WT mice and further exacerbated IOP elevation significantly in *Tg-MYOC^Y437H^* mice. Data are mean ± SEM; *n* = 4–6 in each group; * *P* ≤ 0.05; ** *P* ≤ 0.01; 2-way ANOVA. (**B**) ATG5^fl/fl^ mice (3 months old) were injected with intravitreal injections of Ad5.empty in 1 eye and the contralateral eyes were injected with Ad5.Cre to knock out ATG5 in TM tissues (ATG5_TM_**^–/–^). IOPs were monitored weekly. Loss of ATG5 in TM (ATG5_TM_**^–/–^) significantly elevated IOP compared with control ATG5^fl/fl^ eyes. Data are mean ± SEM; *n* = 4 in each group; * *P* ≤ 0.05; 2-way ANOVA. (**C** and **D**) Ad5.mutant myocilin was injected intravitreally in above ATG5_TM_^+/+^ or ATG5_TM_**^–/–^ eyes. Western blot and densitometric analyses of anterior segment lysates demonstrated that Ad5.mutant myocilin injections increased intracellular myocilin and p62 levels. *n* = 3; unpaired *t* test.

We next examined whether pharmacological or genetic inhibition of autophagy worsens mutant myocilin–induced ocular hypertension. Four-month-old WT or *Tg-MYOC^Y437H^* littermates were given topical ocular CQ eye drops twice daily in 1 eye, and the contralateral eyes were treated with vehicle eye drops for 7 days. CQ treatment significantly increased ocular hypertension in adult *Tg-MYOC^Y437H^* mice, whereas the IOP increase in WT mice was not statistically significant (Figure 4A). We next utilized ATG5^fl/fl^ mice to determine whether loss of ATG5 in TM (ATG5**^–/–^_TM_) leads to ocular hypertension. Ad5 has been shown to exhibit selective tropism for mouse TM (48, 49). We performed intravitreal injections of Ad5.empty in 1 eye; contralateral eyes were injected with Ad5.Cre. IOPs were monitored weekly. Starting from the second week of injection, Ad5.Cre-injected eyes developed significant IOP elevation compared with the contralateral Ad5.empty-injected eyes (Figure 4B). We next performed intravitreal injections of Ad5.expressing mutant myocilin in both eyes. IOP measurement after 2 weeks of injection revealed that mutant myocilin–induced IOP elevation was significantly higher in ATG5**^–/–^_TM_ eyes (Ad5.Cre injected) compared with the contralateral eyes (Supplemental Figure 5). Western blot and densitometric analysis of anterior segment lysates further demonstrated significant knockdown of ATG5 in Ad5.Cre-injected eyes compared with Ad5.empty-injected eyes (Figure 4, C and D). Expression of mutant myocilin led to prominent increases in myocilin, LC3B, and p62 in ATG5**^–/–^_TM_ eyes compared with the contralateral ATG5^fl/fl^ eyes. However, this increase was not found to be statistically significant. We also determined whether Ad5 intravitreal injections exhibit selective tropism for the mouse TM using ROSA 26 transgenic mice expressing tdTomato in which the stop codon was flanked with loxP sites. Ad5Cre intravitreal injections resulted in tdTomato expression in mouse TM (Supplemental Figure 6). These data indicate that autophagy was involved in degradation of WT and mutant myocilin and that genetic or pharmacological inhibition of autophagy led to increased intracellular mutant myocilin accumulation and worsening of mutant myocilin–induced ocular hypertension.

### Depletion of CHOP enhances recognition and degradation of mutant myocilin by autophagy and rescues ocular hypertension in Tg-MYOC^Y437H^ mice.

**Figure 5:**
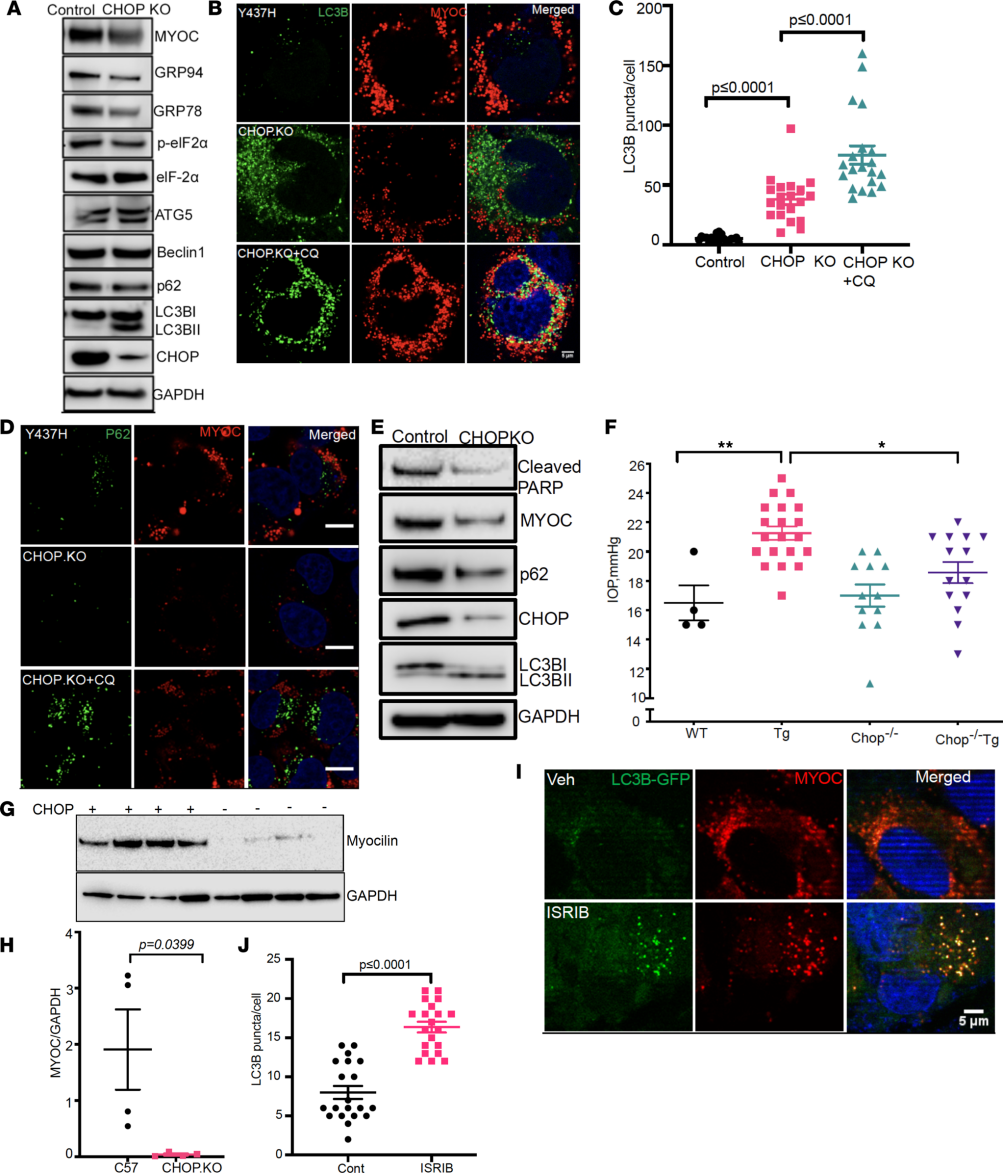
Deletion of CHOP prevents IOP elevation in *Tg-MYOC^Y437H^* mice by promoting autophagic degradation of mutant myocilin. (**A**) TM3 cells stably expressing Y437H mutant myocilin were transfected with CRISPR/Cas9 targeting CHOP. CRISPR/Cas9 targeting CHOP prominently reduced CHOP protein levels and decreased intracellular mutant myocilin. *n* = 3. (**B** and **C**) TM3 cells expressing mutant myocilin were transfected with CRISPR/Cas9 targeting CHOP and lentiviral particles expressing LC3-GFP for 24 hours. Cells were further treated with vehicle or CQ for 12 hours. DsRed and GFP were imaged by confocal microscopy (**B**) and LC3 puncta/cell are shown graphically (**C**). Scale bar: 10 μm. *n* = 3; 1-way ANOVA. (**D**) Immunostaining for p62 demonstrated that reduction of CHOP led to reduced p62. *n* = 3. Scale bar: 10 μm. (**E**) TM3 cells stably expressing mutant myocilin were transfected with CRISPR/Cas9 targeting CHOP and cellular lysates were examined for TM cell death. *n* = 3. (**F**) *Tg-MYOC^Y437H^* mice were crossed with Chop**^–/–^ mice and IOPs were monitored (6 months old). Data are mean ± SEM; *n* = 4 in WT, *n* = 20 in Tg-MYOC, *n* = 12 in CHOP**^–/–^ and *n* = 14 in Chop**^–/–^*Tg-MYOC*, * *P* ≤ 0.05; ** *P* ≤ 0.01, 2-way ANOVA. (**G** and **H**) Western blot and densitometric analyses of anterior segment tissues from WT or Chop**^–/–^ mice injected with Ad.5 expressing mutant myocilin demonstrated significant reduction in mutant myocilin. *n* = 4. Paired *t* test. (**I** and **J**) TM3 cells stably expressing mutant myocilin were transduced with lentiviral particles expressing LC3-GFP and treated either with vehicle or ISRIB (200 nM) for 12 hours. GFP and DsRed color were examined by confocal microscope (**I**) and LC3 puncta counted and represented graphically (**J**). Data are mean ± SEM; *n* = 3; unpaired *t* test.

We have previously shown that mutant myocilin–induced CHOP was associated with loss of TM cells (23). We sought to examine the role of CHOP in mutant myocilin–induced impaired autophagy. TM3 cells stably expressing WT or mutant (Y437H) myocilin were transfected with CRISPR/Cas9 targeting of CHOP, and autophagy markers were examined by Western blot (Figure 5A) and immunostaining (Figure 5, B–D, and Supplemental Figure 7, A and B). Western blot analysis revealed that CRISPR/Cas9 targeting CHOP resulted in a prominent reduction in CHOP protein levels in TM3 cells stably expressing mutant myocilin. Reduction of CHOP (CHOP-KO) was associated with decreased intracellular mutant myocilin and reduced ER stress (Figure 5A) as well as improved autophagy (reduced p62 and increased LC3B II form). CHOP reduction did not alter ATG5 or beclin 1 levels, suggesting that CHOP deletion did not alter autophagy induction. CHOP reduction did not alter myocilin, p62, or LC3B levels in TM3 cells stably expressing WT myocilin (Supplemental Figure 7, A and B). Moreover, reduction of CHOP did not alter MYOC gene expression or secretion (Supplemental Figure 8, A and B). Remarkably, depletion of CHOP significantly enhanced LC3B puncta, which colocalized to mutant myocilin and reduced p62 levels in TM3 cells expressing mutant (Y437H) myocilin (Figure 5, B–D). Inhibition of autophagy via CQ treatment reversed the protective effects of CHOP-KO on mutant myocilin and autophagy. Strikingly, reduction of CHOP reduced mutant (Y437H) myocilin–induced TM cell death, as evident by reduced cleaved PARP and reduced TUNEL-positive cells along with improved autophagy and reduced intracellular mutant myocilin (Figure 5E and Supplemental Figure 9). These data suggest that reduction of CHOP facilitated autophagic degradation of mutant myocilin and prevented TM cell death.

We next determined whether deletion of Chop rescues ocular hypertension in *Tg-MYOC^Y437H^* mice. Chop**^–/–^ mice were crossed with *Tg-MYOC^Y437H^* mice (6 months old) and IOP measurements revealed that *Chop*^+/+^*Tg-MYOC^Y437H^* mice had significantly elevated IOP compared with their *Chop*^+/+^WT littermates (Figure 5F). However, *Chop*^–/–^*Tg-MYOC^Y437H^* mice did not have significantly elevated IOP compared with *Chop*^–/–^ mice. *Chop*^–/–^*Tg-MYOC^Y437H^* mice also exhibited significantly reduced IOP compared with *Chop*^+/+^*Tg-MYOC^Y437H^* mice. We next determined whether deletion of *Chop* improves autophagic degradation of mutant myocilin. We examined autophagy markers in the anterior segment lysates from WT and *Chop*^–/–^ mice injected with Ad5 expressing WT or mutant myocilin. Similar to the results observed with *Chop*^–/–^*Tg-MYOC^Y437H^* mice, intravitreal injections of Ad5.mutant myocilin significantly elevated IOP in WT mice but not in *Chop*^–/–^ mice (Supplemental Figure 10). Western blot and densitometric analysis revealed significantly reduced mutant myocilin levels in *Chop*^–/–^ mice compared with WT littermates (Figure 5, G and H). We further explored whether pharmacological inhibition of the ATF4/CHOP pathway via a small molecule, N,N′-trans-(cyclohexane-1,4-diyl)-bis-(2-(4-chlorophenoxy) acetamide (ISRIB) (50), reduces intracellular mutant myocilin by promoting autophagic degradation of mutant myocilin (Figure 5, I and J). TM3 cells stably expressing mutant (Y437H) myocilin were transduced with lentiviral particles expressing LC3-GFP for 48 hours and then treated with either vehicle or ISRIB (200 nM) for an additional 12 hours. Notably, ISRIB reduced intracellular mutant myocilin and induced LC3 puncta, which colocalized with mutant myocilin, suggesting enhanced cargo recognition and efficient autophagic degradation of mutant myocilin. These data clearly indicate that genetic or pharmacological reduction of CHOP promoted mutant myocilin recognition and its autophagic degradation and rescued mutant myocilin–induced ocular hypertension.

### Tat-beclin 1 peptide or torin 2 promotes autophagic degradation of mutant myocilin and reduces elevated IOP in Tg-MYOC^Y437H^ mice.

**Figure 6:**
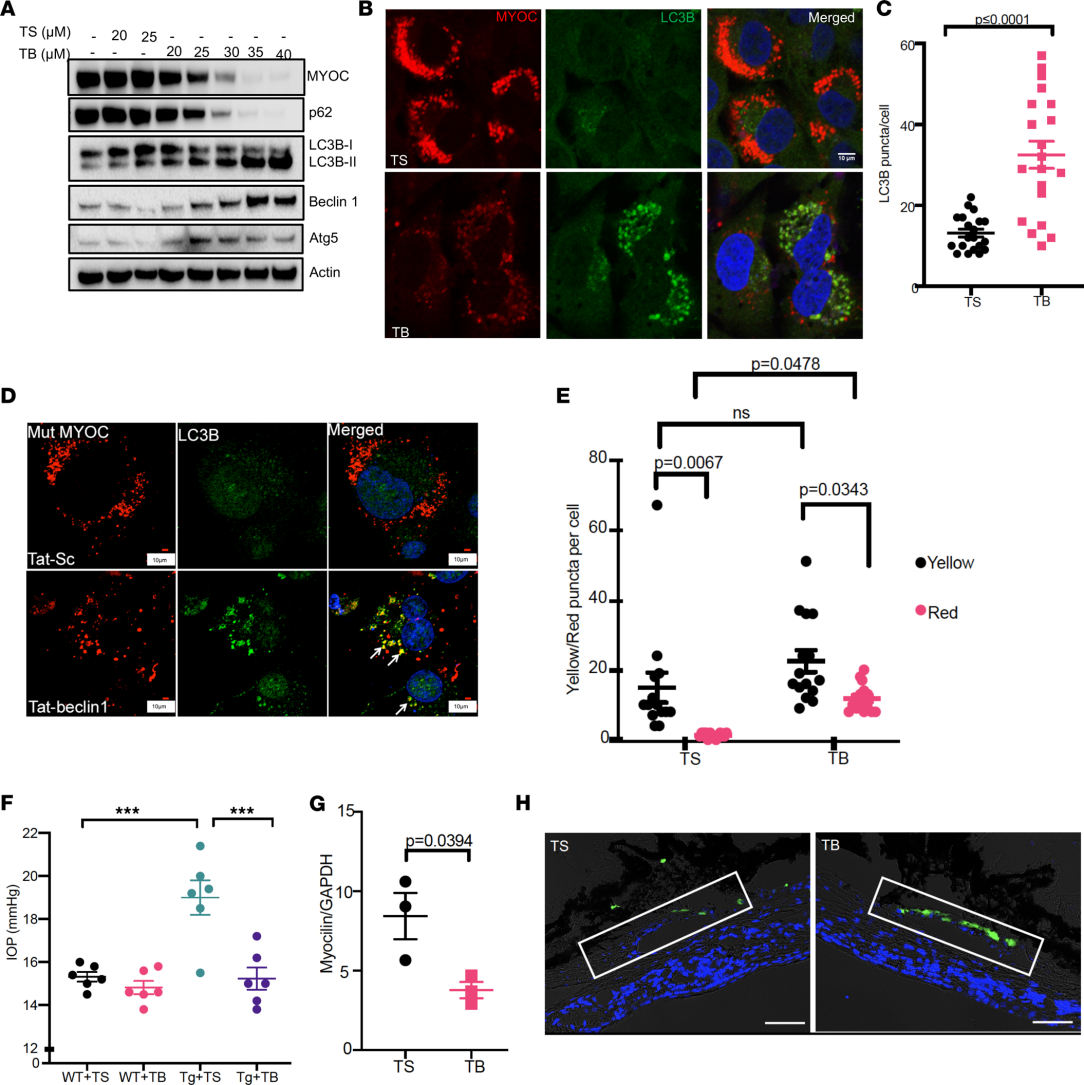
Tat-beclin 1 peptide promotes autophagic degradation of mutant myocilin and reduces elevated IOP in *Tg-MYOC^Y437H^* mice. (**A**) Western blot analysis of TM3 cells stably expressing Y437H mutant myocilin treated with various concentrations of tat-scrambled (TS) or tat-beclin 1 (TB1) peptide. *n* = 3. (**B** and **C**) Immunostaining of LC3B (**B**) and counting of individual LC3B puncta (**C**) in TM3 cells expressing Y437H mutant myocilin treated with TS or TB1 peptide. Scale bar: 10 μm; *n* = 3; unpaired *t* test. (**D**) LC3B and mutant myocilin analysis in human primary TM cells (*n* = 3 cell strains) transduced with Ad5.DsRed-tagged mutant myocilin and treated with TS or TB1. Scale bar: 10 μm. (**E**) Human primary TM cells were transduced with Ad5 expressing mutant myocilin and mRFP-GFP-LC3 and treated with either TS or TB1 peptide for 3 hours. Autophagosome (yellow puncta) and autolysosomes (red puncta) were counted and represented graphically. *n* = 3 cell strains; data are mean ± SEM; 2-way ANOVA. (**F**) Three-month-old WT and ocular hypertensive *Tg-MYOC^Y437H^* littermates were treated with topical ocular TS in 1 eye; the contralateral eyes were treated with TB1 eye drops for 7 days. Nighttime IOP measurement demonstrated that TB1 treatment significantly reduced elevated IOP in *Tg-MYOC^Y437H^* mice. Data are mean ± SEM; *n* = 6 in each group; 2-way ANOVA with Bonferroni’s multiple-comparison test, *P < 0.05, ***P < 0.001. (**G**) Densitometric analyses of Western blot of anterior segment tissue lysates from TS- or TB1-treated mice demonstrated that TB1 peptide treatment significantly reduced myocilin in *Tg-MYOC^Y437H^* mice. Data are mean ± SEM; *n* = 3, paired *t* test. (**H**) LC3-GFP analysis in C57BL/6J mice injected intravitreally with lentiviral particles expressing LC3-GFP and treated with TS (250 μM) or TB1 peptide (250 μM) eye drops for 7 days. The rectangular box indicates the TM region. *n* = 3. Scale bar: 50 μm.

Since expression of mutant myocilin induced impaired autophagic flux in TM cells, we explored whether stimulation of autophagy flux by tat-beclin 1 (TB1) peptide (51) or torin 2 (52) reduces mutant myocilin accumulation in TM cells. TM3 cells stably expressing mutant (Y437H) myocilin were treated with various doses of either tat-scrambled (TS) or TB1 peptide for 3 hours daily for 2 days, and cellular lysates were examined by Western blot analysis (Figure 6A). Starting from 20 μM, TB1 peptide reduced intracellular mutant myocilin in a dose-dependent manner. Concurrently, TB1 peptide also induced autophagy, as evident from increased LC3BII, ATG5, and beclin1 proteins compared with TS-treated TM3 cells. In addition, TB1 peptide reduced p62 levels, further suggesting that correction of mutant myocilin–induced impaired autophagy had occurred. Immunostaining for LC3B demonstrated that TB1 peptide reduced intracellular mutant (Y437H) myocilin accumulation and increased LC3B puncta significantly in TM3 cells and human primary TM cells (*n* = 3 cell strains) compared with TS-treated TM cells (Figure 6, B–D). Strikingly, strong colocalization of mutant (Y437H) myocilin with LC3B puncta was observed in TB1 peptide–treated cells, further suggesting enhanced cargo recognition by autophagy. We next determined whether TB1 peptide improves impaired autophagic flux in primary TM human cells expressing mutant (Y437H) myocilin (Supplemental Figure 11 and Figure 6E). Primary human TM cells (*n* = 3 donor strains) were transduced with Ad5 expressing Y437H mutant myocilin for 48 hours and transduced again with Ad5 expressing RFP-GFP-LC3 for 24 hours, and cells were further treated with TS or TB1 peptide. RFP and GFP were examined using confocal microscopy and LC3 puncta were quantified (Supplemental Figure 11 and Figure 6E). TB1 treatment induced autophagy and improved autophagic flux in TM cells expressing mutant myocilin, as evident from increased autophagic vesicles and a higher number of autolysosomes, respectively.

We have previously shown that the small chemical chaperone sodium 4-phenylbutyrate (PBA) enhances mutant myocilin secretion (23, 53). We examined whether TB1 peptide alters mutant myocilin secretion. Consistent with our studies, TM3 cells stably expressing mutant (Y437H) myocilin demonstrated little or no myocilin in the conditioned medium, and treatment with PBA increased myocilin secretion. However, TB1 peptide did not increase myocilin secretion in the conditioned medium (Supplemental Figure 12). We next examined whether topical ocular TB1 peptide reduces elevated IOP in *Tg-MYOC^Y437H^* mice (Figure 6F). WT and ocular hypertensive *Tg-MYOC^Y437H^* littermates (3 months old) were treated with topical ocular TS peptide in the left eyes; the contralateral right eyes were given topical TB1 peptide twice daily for 7 days. IOP measurements revealed that TB1 peptide did not alter IOP in WT mice significantly compared with TS-treated eyes. However, TB1 peptide significantly lowered elevated IOP in *Tg-MYOC^Y437H^* mice, and IOPs in TB1-treated *Tg-MYOC^Y437H^* mice were similar to that of WT mice. These data clearly indicate that TB1 peptide treatment significantly reduced elevated IOP in *Tg-MYOC^Y437H^* mice. Western blot (Supplemental Figure 13) and densitometric analysis (Figure 6G) demonstrated that TB1 peptide treatment significantly reduced intracellular mutant myocilin accumulation in mouse TM tissue lysates. C57BL/6J mice were injected intravitreally with lentiviral particles expressing LC3-GFP and treated with topical ocular TB1 peptide in the left eyes, and the contralateral right eyes were given TS eye drops (Figure 6H). TB1 peptide treatment increased LC3 puncta in mouse TM, indicating that TB1 peptide can reach and activate autophagy specifically in mouse TM. Western blot analysis of the aqueous humor samples using tat antibody further confirmed the bioavailability of TB1 peptide in the anterior segment tissues of TB1-treated mice (Supplemental Figure 14).

**Figure 7:**
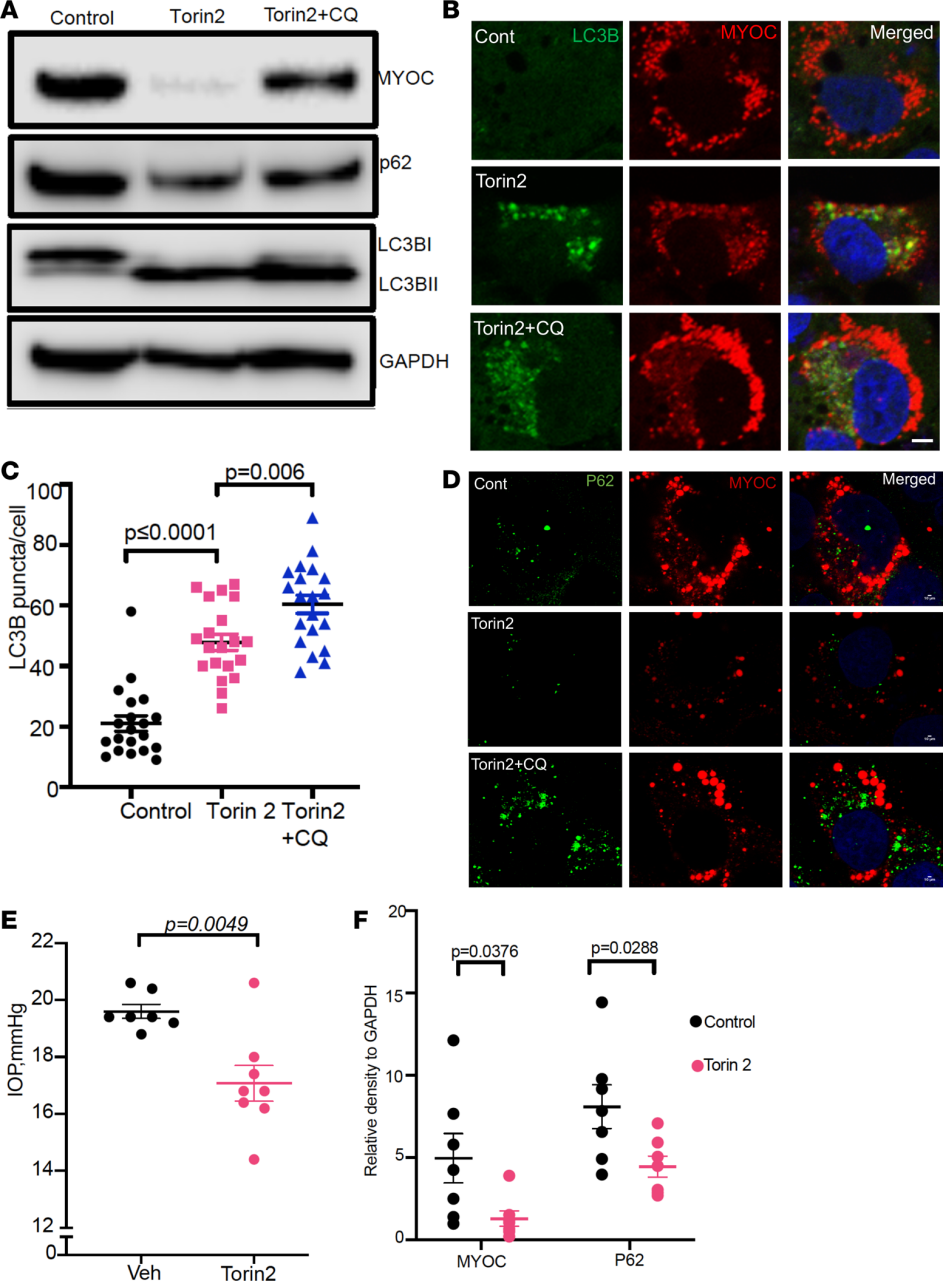
Torin 2 promotes autophagic degradation of mutant myocilin and reduces elevated IOP in *Tg-MYOC^Y437H^* mice. (**A**) TM3 cells stably expressing mutant myocilin were treated with vehicle or torin 2 with or without CQ for 12 hours. Western blot analysis demonstrated that torin 2 reduced intracellular myocilin and increased LC3BII form. These effects were blocked by CQ treatment (*n* = 3). (**B**–**D**) TM3 cells stably expressing mutant myocilin were treated with vehicle or torin 2 along with CQ for 12 hours and fixed cells were stained with LC3B (**B**) and LC3B puncta were counted (**C**). p62 staining is shown in (**D**). Torin 2 treatment stimulated autophagic degradation of mutant myocilin. Scale bar: 10 μm. Data are mean ± SEM; *n* = 3; unpaired *t* test. (**E**) Ocular hypertensive *Tg-MYOC^Y437H^* mice (4 months old) were treated with topical ocular vehicle eye drops in 1 eye; the contralateral eyes were given torin 2 eye drops for 7 days. Torin 2 treatment significantly reduced elevated IOP in *Tg-MYOC^Y437H^* mice. Data are mean ± SEM; *n* = 7–8; paired *t* test. (**F**) Densitometric analyses of Western blot of anterior segment tissue lysates from vehicle- or torin 2–treated mice demonstrated that torin 2 significantly reduced mutant myocilin and p62. Data are mean ± SEM; *n* = 7, *P < 0.05, 1-way-ANOVA.

We next determined whether the chemical activator of autophagy, torin 2, promotes mutant myocilin autophagic degradation and reduces elevated IOP in *Tg-MYOC^Y437H^* mice. TM3 cells expressing mutant (Y437H) myocilin were treated with vehicle or torin 2 with or without CQ. Western blot analysis (Figure 7A) revealed that torin 2 increased LC3BII form and reduced intracellular mutant myocilin and p62 accumulation. Compared with vehicle-treated TM3 cells expressing mutant myocilin, torin 2–treated TM3 cells expressing mutant myocilin exhibited a prominent and significant increase in LC3B colocalization with mutant myocilin, suggesting enhanced cargo recognition (Figure 7, B and C). Torin 2 also reduced p62 levels in TM3 cells expressing mutant (Y437H) myocilin (Figure 7D). Consistently, treatment with CQ abrogated protective effects of torin 2 on myocilin and autophagy.

We next explored whether topical ocular torin 2 treatment reduces elevated IOP in *Tg-MYOC^Y437H^* mice (Figure 7E). Ocular hypertensive *Tg-MYOC^Y437H^* mice were given topical ocular eye drops of vehicle in 1 eye, and the contralateral eyes were treated with torin 2 twice daily for 7 days. IOP measurements revealed that torin 2 significantly reduced IOP in *Tg-MYOC^Y437H^* mice. Western blot (Supplemental Figure 15) and densitometric analysis (Figure 7F) revealed that torin 2 treatment significantly reduced myocilin and p62 accumulation in mouse TM tissue lysates. Liquid chromatography–mass spectrometry analysis of the aqueous humor also revealed the bioavailability of torin 2 in the mice treated with torin 2 (Supplemental Figure 16). In another cohort of mice, we treated ocular hypertensive *Tg-MYOC^Y437H^* mice with torin 2 eye drops for a week and monitored IOPs weekly (Supplemental Figure 17). Starting from the first week of treatment, torin 2 eye drops reduced IOP significantly in *Tg-MYOC^Y437H^* mice. Interestingly, IOPs remained significantly lower for another 3 weeks (although torin 2 treatment was stopped after 1 week) and IOP returned to baseline in *Tg-MYOC^Y437H^* mice that had elevated IOP after 4 weeks of treatment. These data indicate that induction of autophagy by torin 2 reduced mutant myocilin accumulation and significantly lowered elevated IOP in *Tg-MYOC^Y437H^* mice over an extended period of time.

## Discussion

Mutations in myocilin are the leading known genetic cause of POAG (2, 13). Protein misfolding caused by mutant myocilin leads to chronic ER stress, which is associated with TM dysfunction/loss, leading to IOP elevation in mice (23). However, it is not understood how chronic ER stress leads to TM dysfunction/loss. Here, we report that mutant myocilin activated autophagy, but it was functionally impaired. Genetic or pharmacological inhibition of autophagy exacerbated intracellular mutant myocilin accumulation and worsened ocular hypertension in *Tg-MYOC^Y437H^* mice. Impaired autophagy was associated with chronic ER stress in TM cells in vitro and in vivo. Remarkably, *Chop* deletion rescued ocular hypertension in *Tg-MYOC^Y437H^* mice and prevented TM cell death by promoting autophagic degradation of mutant myocilin. Furthermore, stimulation of autophagy via TB1 peptide or torin 2 reduced IOP elevation in *Tg-MYOC^Y437H^* mice by promoting autophagic degradation of mutant myocilin. Together, our findings indicate that correction of autophagy promoted efficient recognition and degradation of misfolded myocilin by autophagy and rescued a mouse model of myocilin-associated POAG.

Expression of mutant myocilin activated autophagy, as evident from increased beclin 1 and ATG5. However, increased LC3BI form, increased autophagosome, and p62 accumulation indicated that autophagy progression was impaired. Consistent with a previous study (42), we also observed that autophagy was involved in degradation of WT or mutant myocilin, as evident from increased intracellular WT or mutant myocilin when autophagy was inhibited. However, there was minimal or no colocalization of mutant myocilin with LC3B puncta in TM cells expressing mutant myocilin. In addition, LC3B puncta were also scarcely present in mutant myocilin–expressing cells despite prominent accumulation of misfolded myocilin. These data indicate that autophagy was impaired, and perhaps induced autophagy failed to recognize misfolded mutant myocilin, which might further overwhelm protein degradation pathways. A previous study by Porter et al. demonstrated that autophagy is dysregulated in glaucomatous TM cells (likely to be from nonmyocilin glaucoma patients) (54). Moreover, impaired autophagic flux was observed in TM tissues of DBA/2J mice, a model for human pigmentary glaucoma (55). Together, these studies further support the notion that impaired autophagy may play a critical role in TM dysfunction and IOP elevation in general cases of POAG and is not limited to myocilin-associated POAG.

The role of basal autophagy in IOP homeostasis has not been previously explored, to our knowledge. We showed that loss of ATG5 in TM led to significant IOP elevation, suggesting that a basal level of autophagy is required for IOP maintenance. We also showed that impaired autophagy was associated with IOP elevation in the mouse model of myocilin POAG. Inhibition of autophagy via CQ treatment or loss of ATG5 in the TM exacerbated IOP elevation in *Tg-MYOC^Y437H^* mice. These findings suggest a critical role for autophagy in IOP homeostasis.

Induction of CHOP is associated with several diseases, including cancer, diabetes, neurodegenerative disorders, and fibrosis (56, 57). We have shown previously that induction of CHOP is associated with TM cell dysfunction/loss and IOP elevation in *Tg-MYOC^Y437H^* mice (23). CHOP levels were found to be significantly increased in TM tissues from general POAG donor eyes (58). In addition, deletion of *Chop* rescued a mouse model of glucocorticoid-induced glaucoma (59). These studies linked the role of CHOP to the pathophysiology of general glaucoma. However, the exact mechanism of how induction of CHOP leads to cell death is not completely understood. In the present study, we propose that induction of CHOP by mutant myocilin plays an important role in dysfunctional autophagy. Consistent with this, we showed that reduction of CHOP corrected autophagy and promoted autophagic degradation of mutant myocilin, in addition to rescuing ocular hypertension in *Tg-MYOC^Y437H^* mice. Reduction of CHOP did not alter mRNA expression of mutant myocilin, suggesting that myocilin reduction is a posttranscriptional effect. Disease-causing myocilin mutants are secretion incompetent, and improving secretion of mutant myocilin via sodium 4-phenylbutyate rescues glaucoma in *Tg-MYOC^Y437H^* mice (53). Contrary to this, CHOP deletion or stimulation of autophagy did not improve mutant myocilin secretion. These findings further support our conclusion that reduction of CHOP reduces mutant myocilin via promoting autophagic degradation of mutant myocilin. We have recently shown that chronic ER stress induced ATF4, and CHOP promoted increased protein synthesis, which leads to ER client protein load (43). It is therefore possible that chronic ER stress–induced protein synthesis and ER client protein load may overwhelm autophagy. CHOP deletion may reduce protein synthesis and ER client load, thus allowing autophagy to function normally. We observed that CHOP reduction did not alter ATG5 or beclin1 protein levels, suggesting that CHOP deletion did not further induce autophagy, but reduction of CHOP was associated with improved autophagy. Specifically, CHOP deletion enhanced cargo (mutant myocilin) recognition by autophagy and facilitated its autophagic degradation.

Knockdown of GRP94 has been shown to activate autophagy, which may be effective in removal of abnormal myocilin accumulation (60). However, GRP94 is a component of the GRP78 chaperone system in protein processing and has prosurvival properties (61). Knockdown of GRP94 may not be a good candidate for long-term treatment since it can affect protein folding of other secreted proteins. In contrast, more distal UPR cell death effectors such as CHOP provide ideal candidates for long-term targeted treatment.

It is plausible that autophagy activation by mutant myocilin is not robust and fails to recognize mutant myocilin aggregates efficiently. This is evident from lack of distinct LC3-positive autophagic vesicles that colocalized with mutant myocilin. Consistent with this, robust induction of autophagy via the TB1 peptide or torin 2 recognized and degraded mutant myocilin efficiently. TB1 peptide or torin 2 also corrected impaired autophagy and reduced IOP elevation in *Tg-MYOC^Y437H^* mice. Although the TB1 peptide has been shown to decrease polyglutamine protein aggregates in vitro (51), the efficacy of the TB1 peptide in animal models of protein misfolding has not been tested. We showed in vivo effectiveness of the TB1 peptide on reduction of elevated IOP in *Tg-MYOC^Y437H^* mice.

Autophagy is known to play either a prosurvival or prodeath role depending on the context (62, 63). Loss of TM cells is associated with POAG (11). We and others have shown that expression of mutant myocilin leads to TM cell death (21, 23, 25), which is associated with induction of CHOP. It is therefore indicated that impaired autophagy induced by CHOP plays a key role in TM cell death. Consistent with this, our studies clearly showed that correction of impaired autophagy via deletion of CHOP was a prosurvival mechanism and reduced mutant myocilin–induced TM cell death, as evident from reduced cleaved PARP.

In conclusion, our data indicate that expression of mutant myocilin led to impaired autophagy, which was associated with induction of CHOP. Reduction of CHOP or treatment with autophagy inducers corrected impaired autophagy flux, which resulted in better cargo recognition, promoting efficient autophagic degradation of mutant myocilin and reducing elevated IOP in the mouse model of glaucoma. These studies elucidate the interplay between chronic ER stress and autophagy in IOP elevation and further provide alternate treatment strategies for myocilin-associated POAG by targeting these pathologies.

## Methods

### Antibodies and reagents.

Antibodies were purchased from the following sources: LC3B (MilliporeSigma, L7543), P62 (MBL International, PM045), MYOC (Santa Cruz Biotechnology, [N15]-sc-21243), CHOP (Novus Biologicals, 13172), Beclin 1 (MBL International, PD017), and ATG5 (Cell Signaling Technology, D5F5U). To probe mouse anterior segment tissues, we used a different myocilin antibody (Abnova, H00004653-MO1). Adenoviral vectors expressing empty DsRed-tagged WT or mutant myocilin and LC3-RFP-GFP under the control of CMV promoter were made by ViraQuest Inc. Adenoviral vectors expressing empty, WT, or mutant myocilin (no tag) were obtained from the Viral Vector Core Facility at the University of Iowa. The reagents were purchased from the following sources: Tat-Beclin 1(D11) (Novus Biologicals, NBP2-49888), Tat-Beclin 1 (L11) scrambled (Novus Biologicals, 49887), Torin 2 (MilliporeSigma, SML 1224), chloroquine diphosphate (Life Technologies, P36239), and Opti-MEM (Thermo Fisher Scientific, 11058021).

### Animals.

CHOP-KO mice were purchased from The Jackson Laboratory. ATG5^fl/fl^ mice on a pure C57BL/6J background were provided by Noboru Mizushima (The University of Tokyo, Tokyo, Japan), and these mice were received from Thomas Ferguson’s lab (Washington University School of Medicine, St. Louis, Missouri, USA). The development and characterization of transgenic mice expressing mutant myocilin under the control of CMV promoter (*Tg-MYOC^Y437H^*) was reported previously (23, 53). All strains of mice were on a pure C57BL/6J background. Animals were maintained under 12-hour light/12-hour dark cycles and had access to food and water ad libitum. For most studies, we utilized 3- to 4-month-old WT and *Tg-MYOC^Y437H^* mice because we have observed the onset of ocular hypertension at this stage (23). For *Chop*^–/–^ studies, we performed IOP measurements at 6 months of age to determine whether deletion of Chop prevents ocular hypertension for longer duration of time.

### IOP measurements.

Daytime and nighttime IOPs were measured using a TonoLab rebound tonometer (Colonial Medical Supply) under isoflurane anesthesia as previously described (64, 65). For measuring nighttime IOPs, mice were kept in the dark for 7 hours (3 pm to 10 pm), and IOPs were measured in the dark using dim red lights. Six individual IOP measurements were obtained in a masked manner and averaged to obtain the final IOP value for each eye. Daytime IOPs were measured between 9 am and 11 am. Conscious (without anesthesia) IOPs were measured in Figure 2 as described previously (64). For all other experiments, nighttime IOPs under isoflurane anesthesia were measured.

### Cell culture and stable cell production.

Human primary TM cells were isolated from different donor eyes (*n* = 5) without any history of glaucoma and are characterized as described previously (66, 67). Both primary and transformed TM3 cells were cultured in DMEM supplemented with 10% FBS (Hyclone Laboratories), 2 mM l-glutamine (Thermo Fisher Scientific), penicillin (10,000 units/mL; Thermo Fisher Scientific), and streptomycin (10 μg/mL; Thermo Fisher Scientific). Cells were maintained in a humidified incubator at 5% CO_2_ and 37°C as described previously (68). TM3 cells stably expressing DsRed-tagged WT or mutant (Y437H, G364V, and Q368X) myocilin were generated by transfecting with plasmid expressing DsRed-tagged WT or mutant (Y437H, G364V, and Q368X) myocilin containing a G418 selection. The stable cell colonies were then selected using G418 (0.6 mg/mL; Gibco, Life Technologies) containing media and stable cells were maintained in the same medium as described previously (43, 68).

### CHOP knockdown in TM3 cells.

TM3 cells expressing WT or mutant myocilin were transfected with plasmid expressing CRISPR/Cas9 targeting CHOP for 24 hours and cells were selected using puromycin antibiotics. To inhibit autophagy, puromycin-selected cells were treated with vehicle or CQ for 12 hours and cellular lysates or fixed cells were subjected to Western blot analysis and immunostaining, respectively.

### Treatment of cells and mice.

TM cells stably expressing mutant myocilin were treated with TB1(D11) or TS peptide for 3 hours daily for 2 days in acidified Opti-MEM (0.15% HCl containing Opti-MEM). TM cells were treated with vehicle (DMSO) or 10 μM torin 2 (dissolved in DMSO) for 12 hours to induce autophagy. TM3 cells were treated with vehicle or CQ (50 μM) for 12 hours or BA1 (200 nM) for 6 hours to inhibit autophagy. WT and *Tg-MYOC^Y437H^* littermates were treated with topical ocular TB1(D11) peptide (250 μM dissolved in PBS) eye drops in 1 eye, and the contralateral eyes were treated with TS peptide (250 μM dissolved in PBS) twice daily for a week. Topical ocular torin 2 (100 μM in PBS) eye drops were given in 1 eye; the contralateral eyes were applied with PBS twice daily. To determine whether TB1 peptide induces autophagy in mouse TM, C57BL/6J mice were injected intravitreally with lentiviral particles expressing LC3-GFP (2 μL) in both eyes. One week later, 1 eye was treated with TB1 peptide (250 μM) eye drops, and the contralateral eyes were given TS (250 μM) eye drops for 1 week. Mouse anterior segment sections were examined for LC3-GFP expression. The effects of autophagy inhibition in mice were studied by applying CQ (10 mM; dissolved in PBS) eye drops twice daily for a week. The contralateral eyes were treated with vehicle (PBS). IOPs were monitored before and after treatment weekly. For ISRIB treatment, TM3 cells stably expressing mutant myocilin were transduced with lentiviral particles expressing LC3-GFP (generated in-house) for 48 hours. TM3 cells were then treated with ISRIB (200 nM) or corresponding amount of DMSO (vehicle) for 12 hours. GFP and DsRed expression were examined by confocal microscope (Leica SP8).

### Immunostaining.

Enucleated mouse eyes were fixed in 4% paraformaldehyde (PFA) overnight and embedded in either OCT or paraffin. Ten-micron OCT sections were made using a cryotome (Leica Biosystems Inc.). Tissue sections were allowed to dry at room temperature and permeabilized using 0.1% Triton X-100 in PBS for 10 minutes. Sections were then blocked with 10% goat serum for 2 hours and incubated with primary antibody (1:100) in blocking buffer overnight at 4°C. Sections were washed 3 times with 1× PBS and incubated with the appropriate Alexa Fluor secondary antibody (1:200; Invitrogen). Slides were then washed 3 times with 1× PBS and mounted with DAPI mounting media (VECTASHIELD Antifade Mounting Medium, Vector Laboratories). Images were taken using a Leica SP8 confocal microscope. For immunostaining of paraffin-embedded tissues, 5 μm paraffin sections were prepared, deparaffinized, and subjected to antigen retrieval in citrate buffer (pH 6). Slides were then blocked in 10% goat serum containing 0.1% Triton X-100 for 2 hours and incubated with primary and secondary antibodies as described above and previously (69, 70). For immunostaining of TM cells, cells were fixed in 4% PFA for 15 minutes, permeabilized with 0.1% Triton X-100 in PBS for 10 minutes, and stained with the appropriate antibodies as described above.

### Western blot analysis.

Cells or mouse anterior segments (containing entire TM and parts of ciliary body and iris) were lysed in RIPA lysis buffer, and cell debris was removed by centrifugation at 12,000*g* for 10 minutes. Next, 20–30 μg of lysates were loaded on denaturing 4%–12% gradient polyacrylamide readymade gels (NuPAGE Bis-Tris gels, Life Technologies) and run with 1× MES SDS running buffer (NuPAGE, Invitrogen) as described previously (23, 59). The separated proteins were then transferred onto PVDF membranes. The blots were blocked with 10% nonfat dry milk prepared in 1× PBST for 1 hour and incubated with primary antibodies (1:1000) overnight on a rotating shaker at 4°C. The membranes were washed 3 times for 5 minutes each with 1× PBST and incubated with appropriate HRP-conjugated secondary antibodies. After 3 washes with 1× PBST, blots were developed with ECL detection reagents (SuperSignal West Femto Maximum Sensitivity Substrate; Life Technologies) using a LI-COR Odyssey Fc image system. Because mouse anterior segment lysates contain non-TM tissues, we observed variability in Western blot results. See complete unedited blots in the supplemental material.

### Measuring autophagic flux in cells.

The autophagic flux was measured using an mRFP-GFP-LC3 tandem fluorescent protein-quenching assay. This assay relies on the principle that the fluorescence of GFP (pKa = 5.9) is quenched in acidic compartments, whereas fluorescence of mRFP (pKa = 4.5) is stable in acidic compartments like lysosomes. The mRFP-GFP-LC3 exhibits both red and green (yellow in merged image) fluorescence, indicating autophagosomes. Expression of red color alone indicates autolysosomes. Measuring red/yellow puncta per cell gives the rate of autophagic flux (35). More red/yellow puncta represents more autophagic flux. The primary human TM cells (*n* = 3 cell strains) were plated in 8-well chamber slides (Nunc Lab-Tek Chamber Slide system) and transduced with adenoviral vectors expressing WT and mutant (Y437H) myocilin for 48 hours. Primary human TM cells expressing WT and mutant myocilin were retransduced with adenoviral vectors expressing the mRFP-GFP-LC3 construct for another 24 hours. Cells were then fixed in 2% PFA for 5 minutes and fluorescent images were immediately taken using a Leica SP8 confocal microscope. The numbers of red and yellow puncta were counted per cell and represented graphically.

### Real-time PCR analysis.

MYOC expression in TM3 cells stably expressing mutant myocilin treated with or without CRISPR/Cas9 targeting CHOP were analyzed using quantitative real-time PCR. RNeasy Mini Kit (Qiagen) was used to isolate total RNA following the manufacturer’s instructions. Isolated RNA was analyzed for purity and concentration using a NanoDrop 2000 (Thermo Fisher Scientific). The SuperScript VILO cDNA synthesis kit (Thermo Fisher Scientific) was used to synthesize cDNA from isolated RNA. Quantitative PCR was performed using a BioRad CFX96 thermocycler and 2× SsoAdvanced SYBR Green Supermix. The PCR conditions were used as follows: An initial 95°C 60-second incubation was followed by 40 cycles at 95°C for 60 seconds, 60°C for 45 seconds, and 72°C for 45 seconds and completed with a dissociation curve. The following PCR primer pairs were used: hMYOC forward: TACAGGCAATGGCAGAAGGA, hMYOC reverse: GAAAGCAGTCAAAGCTGCCT, hACTB forward: GCCGCCAGCTCACCAT, hACTB reverse: AATCCTTCTGACCCATGCCC. The CFX96 thermocycler generated Ct values. The Ct method was used to calculate myocilin mRNA expression levels versus vehicle using a β-actin internal control as previously reported (65, 68).

### Statistics.

Data are expressed as the mean ± SEM (*n* ≥ 3).The experiments were repeated in 3 independent batches whenever representative results are shown. Primary human TM cells from 5 different donor eyes were utilized. GraphPad Prism 8 was used to determine statistical significance. Data were analyzed using 2-tailed Student’s *t* test for comparison between 2 groups or 1-way or 2-way ANOVA with Tukey’s post hoc test for comparisons between more than 2 groups. A *P* value less than 0.05 was considered statistically significant.

### Study approval.

All experimental procedures were conducted in accordance with and adherence to the Association for Research in Vision and Ophthalmology Statement for the Use of Animals in Ophthalmic and Vision Research. The experimental protocol was approved by the IACUC of the University of North Texas Health Science Center (Protocol IACUC-2018-0032).

## Author contributions

RBK and GSZ designed research studies, analyzed data, provided reagents, and wrote the manuscript. PM, CK, CCS, SP, and VCS assisted in conducting key experiments, provided reagents, and assisted in manuscript preparation. BL provided reagents and assisted in key experiments. All authors discussed the results and implications and commented on the manuscript at all stages.

## Supplementary Material

Supplemental data

## Version 1 02/04/2021

In-Press Preview

## Version 2 03/08/2021

Electronic publication
