# Supplementary material for: Autophagy stimulation reduces ocular hypertension in a murine glaucoma model via autophagic degradation of mutant myocilin
Source: JCI Insight. 2021 Mar 8;6(5):e143359. doi: 10.1172/jci.insight.143359 (PMC8021112; doi:10.1172/jci.insight.143359)

Supplementary Figures

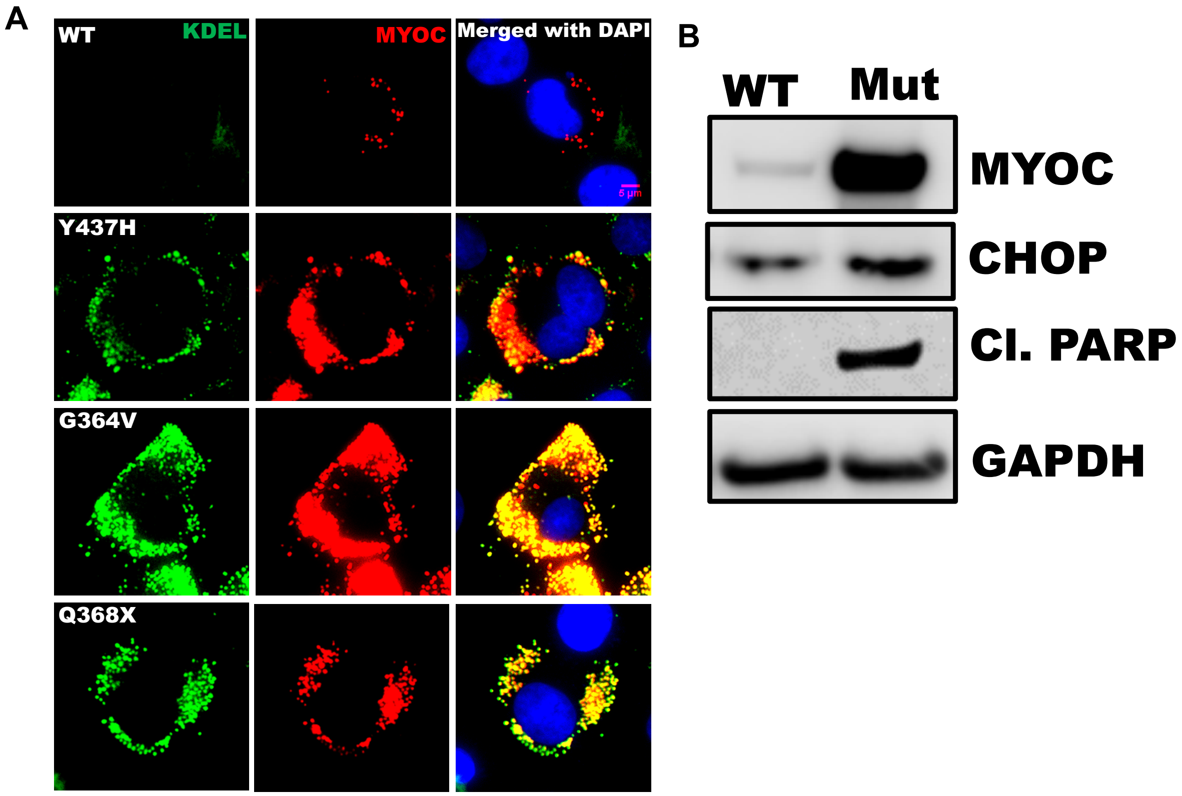

SI.1: Mutant myocilin accumulates in the endoplasmic reticulum (ER), induces ER stress and leads to TM cell death. A) TM3 cells stably expressing DsRed-tagged WT or individual mutants of myocilin (Y437H, G364V, and Q368X) were stained with the ER marker, KDEL. A prominent colocalization of mutant myocilin with KDEL indicates mutant myocilin accumulates in the ER. N=3 technical replicates. Scale bar is 5µm. **B)** Western blot analysis of myocilin, CHOP and pro-apoptotic markers (cleaved PARP) in the lysates of TM3 cells expressing WT or mutant MYOC (Y437H). Expression of mutant myocilin induced chronic ER stress marker, CHOP, which is associated with cleaved PARP indicating that mutant myocilin and induction of CHOP is associated with TM cell death. N=2 technical replicates.

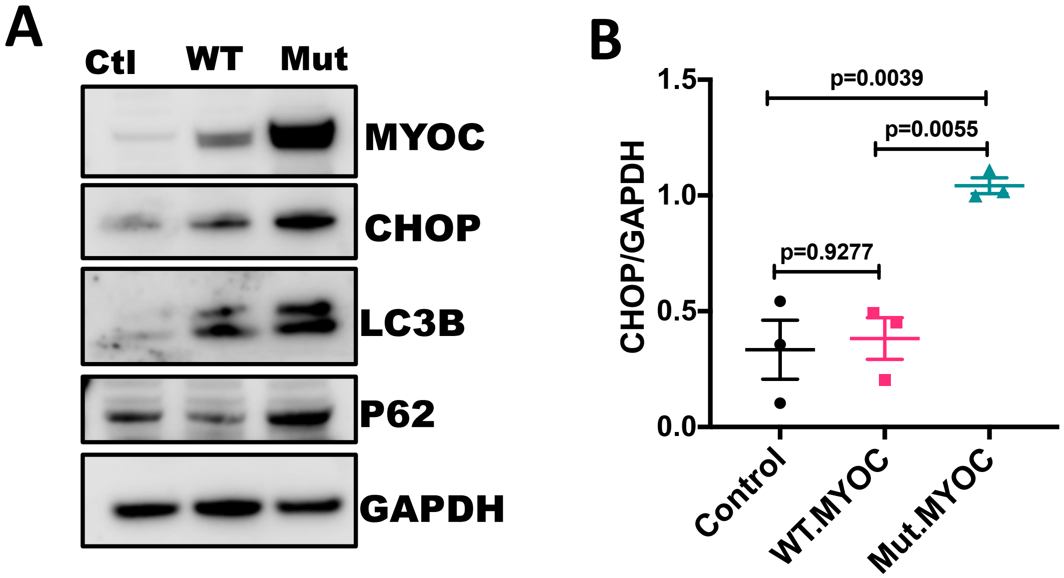

SI.2: Expression of mutant myocilin leads to impaired autophagy, which is associated with induction of CHOP. A) TM3 cells expressing WT or Y437H mutant of myocilin were cultured and cell lysates were subjected to Western blot analysis of the autophagy markers LC3B and P62. Both LC3B and P62 levels were increased in mutant myocilin expressing cellular lysates compared to WT myocilin, clearly indicating an impairment of autophagy. **B)** Densitometric analysis of Western blots for CHOP indicates that impaired autophagy was associated with significant induction of CHOP in TM3 cells expressing mutant myocilin. N=3 technical replicates. 1-way ANOVA.

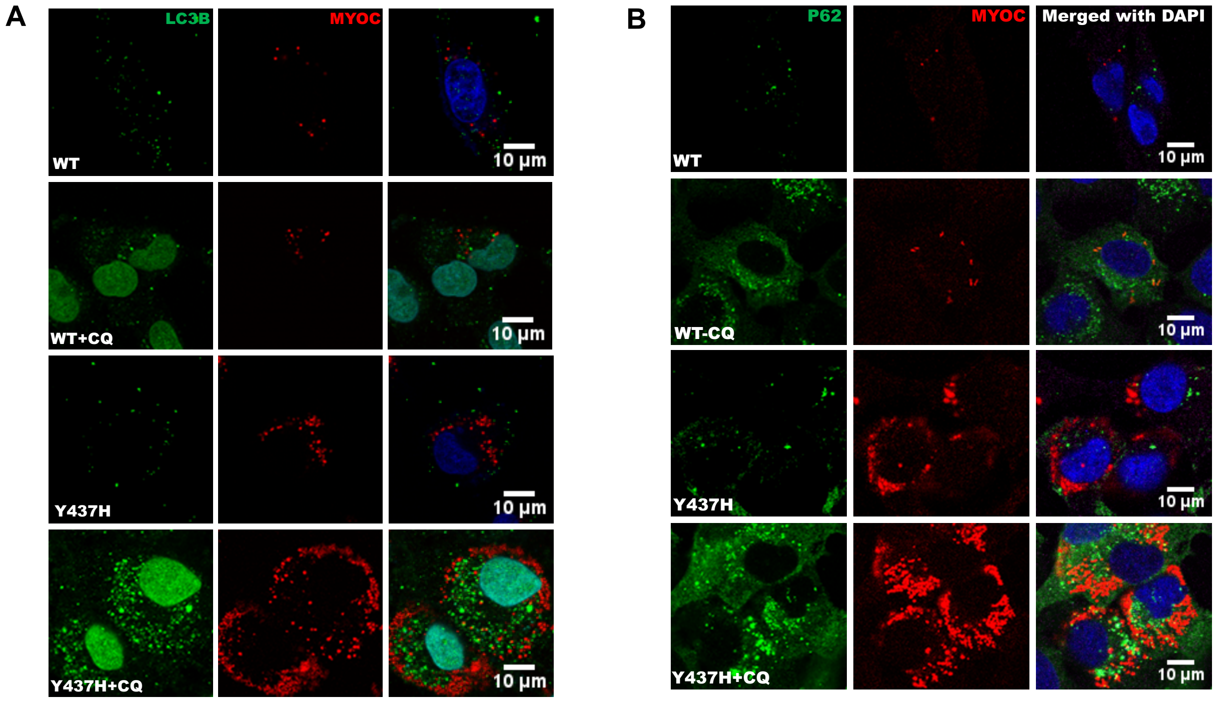

SI.3: Inhibition of autophagy exacerbates myocilin accumulation in TM cells. TM3 cells stably expressing DsRed-tagged WT or mutant (Y437H) myocilin treated with either vehicle or chloroquine (CQ) for 12 hrs. TM cells were stained with LC3B (**A**) and p62 (**B**). Chloroquine treatment resulted in increased myocilin (WT and mutant), LC3B puncta, and P62 levels. N=2 replicates. Scale bar is 10µm.

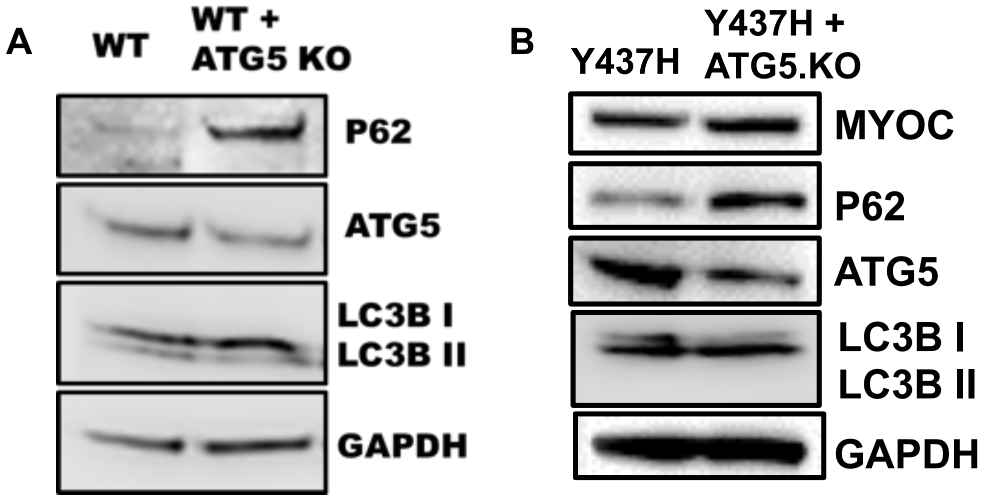

SI.4: Knockdown of ATG5 exacerbates WT and mutant myocilin accumulation in TM3 cells. ATG5 was transiently knocked down in TM3 cells stably expressing WT (**A**) or mutant myocilin (**B**) and cellular lysates were subjected to Western blot analysis of autophagy markers. ATG5 knockdown reduced ATG5 protein and inhibited autophagy as evident from increased LC3B and P62 levels. Moreover, ATG5 knockdown further increased intracellular mutant myocilin accumulation in TM3 cells. N=3 replicates.

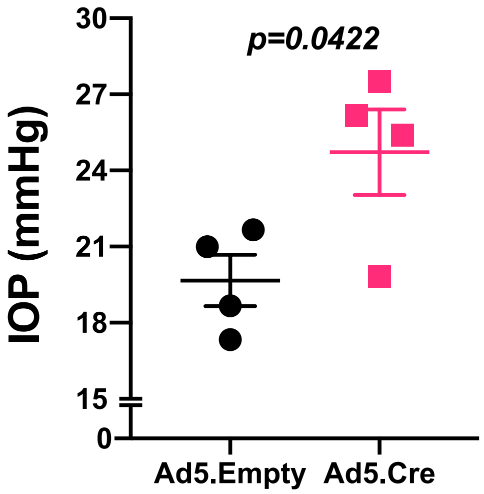

SI.5: Inhibition of autophagy further worsens myocilin-induced IOP elevation in mice. $ATG5^{F/F}$ mice (4-month old) were injected intravitreally with either Ad5.Empty in one eye while the contralateral eyes were injected with Ad5.Cre. 5-weeks later, both eyes were again injected intravitreally with Ad5 expressing mutant myocilin and IOPs were measured after 2 weeks of injections. Mutant myocilin significantly increased IOP elevation in Ad5.Cre ($ATG5^{-/-}{}_{TM}$) injected mice compared to Ad5.Empty ($ATG5^{+/+}{}_{TM}$) injected mice. Data are mean ± SEM; N=4 each; paired t-test.

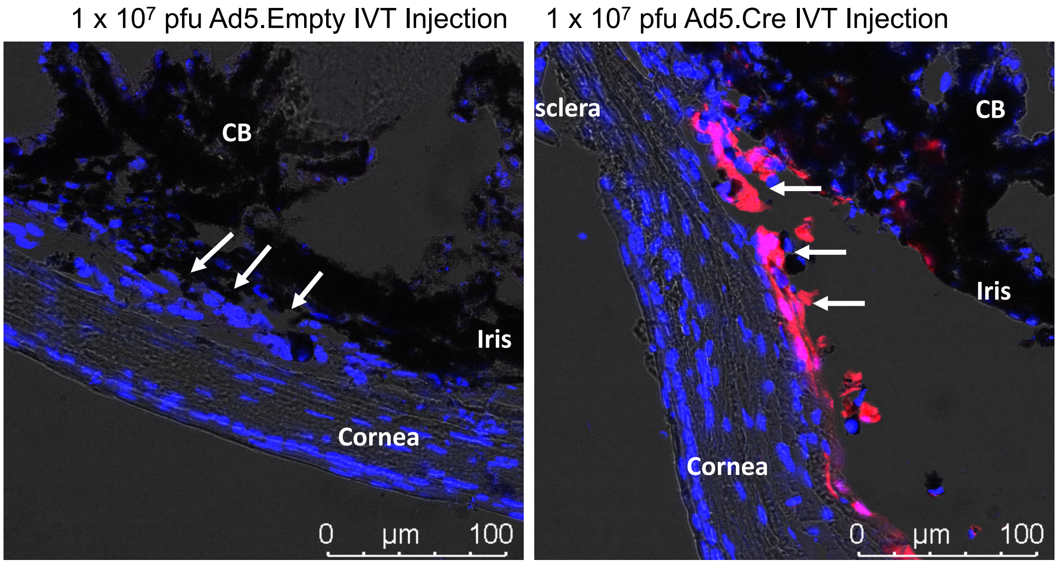

SI.6: Intravitreal injection of Ad5.Cre induces tdTomato in mouse TM. B6.Cg-*Gt(ROSA)26Sor*$^{tm9(CAG\text{-}tdTomato)Hze}$/J mice (Stock number 007909; Jackson labs) were injected intravitreally with either Ad5.Empty in one eye while the contralateral eyes were injected with Ad5.Cre (n=3 mice). 2-weeks later, mouse eyes were fixed and examined for tdTomato expression using confocal microscopy. Arrows indicate TM. N=3 mice. Scale bar is 100μm.CB= ciliary body.

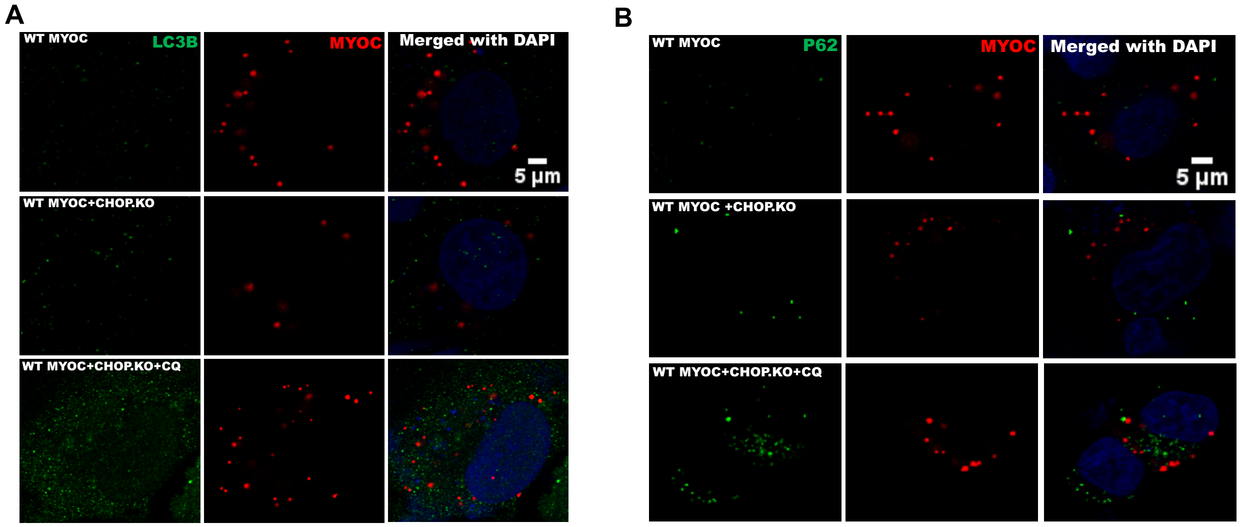

SI.7: Reduction of CHOP does not alter WT MYOC levels in TM3 cells stably expressing WT MYOC. TM3 cells stably expressing WT myocilin were transfected with either vehicle (transfection reagents) or CRISPR-Cas9 targeting CHOP and then treated with or without chloroquine (CQ) for 12 hrs. TM3 cells were then stained with LC3B (**A**) or P62 (**B**). The CHOP knockdown did not alter intracellular WT myocilin, LC3B puncta and p62 levels compared to their respective controls. N=2 replicates. Scale bar is 5µm.

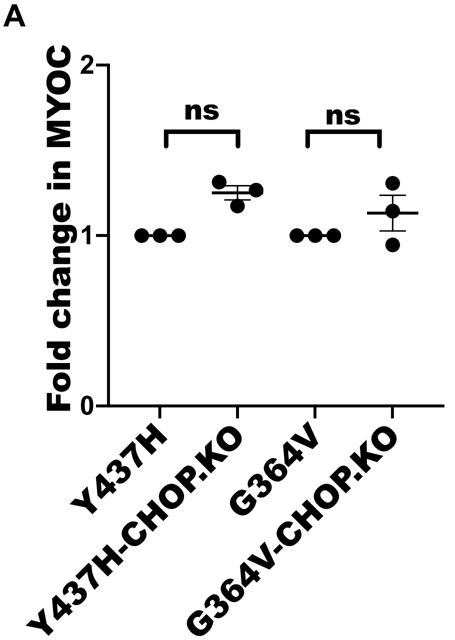

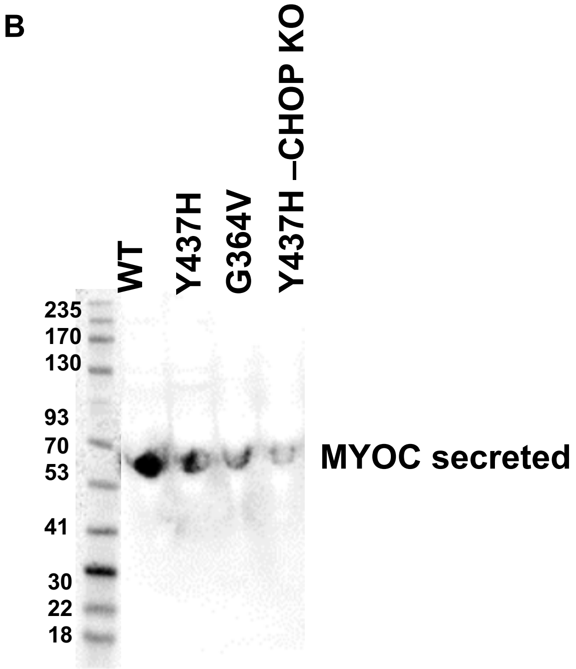

SI.8: Reduction of CHOP does not alter MYOC gene expression or secretion. A) QPCR analysis of MYOC mRNA expression in TM3 cells stably expressing mutant myocilin (Y437H or G364V) with or without CHOP knockdown by CRISPR-Cas9 revealed that CHOP knockdown does not significantly alter myocilin mRNA expression compared to their respective controls. Data are presented as mean ± SEM; N=3; 2-way ANOVA. **B)** TM3 cells stably expressing WT or mutant myocilin (Y437H or G364V) were transduced with or without CRISPR-Cas9 targeting CHOP for 24hrs. The conditioned medium was subjected to Western blot analysis of myocilin. TM3 cells stably expressing WT myocilin secreted myocilin. However, TM3 cells stably expressing mutant myocilin (Y437H or G364V) demonstrated inhibition of myocilin in the conditioned medium. Moreover, CHOP reduction did not improve myocilin secretion in TM3 cells expressing Y437H mutant myocilin. N=2 technical replicates.

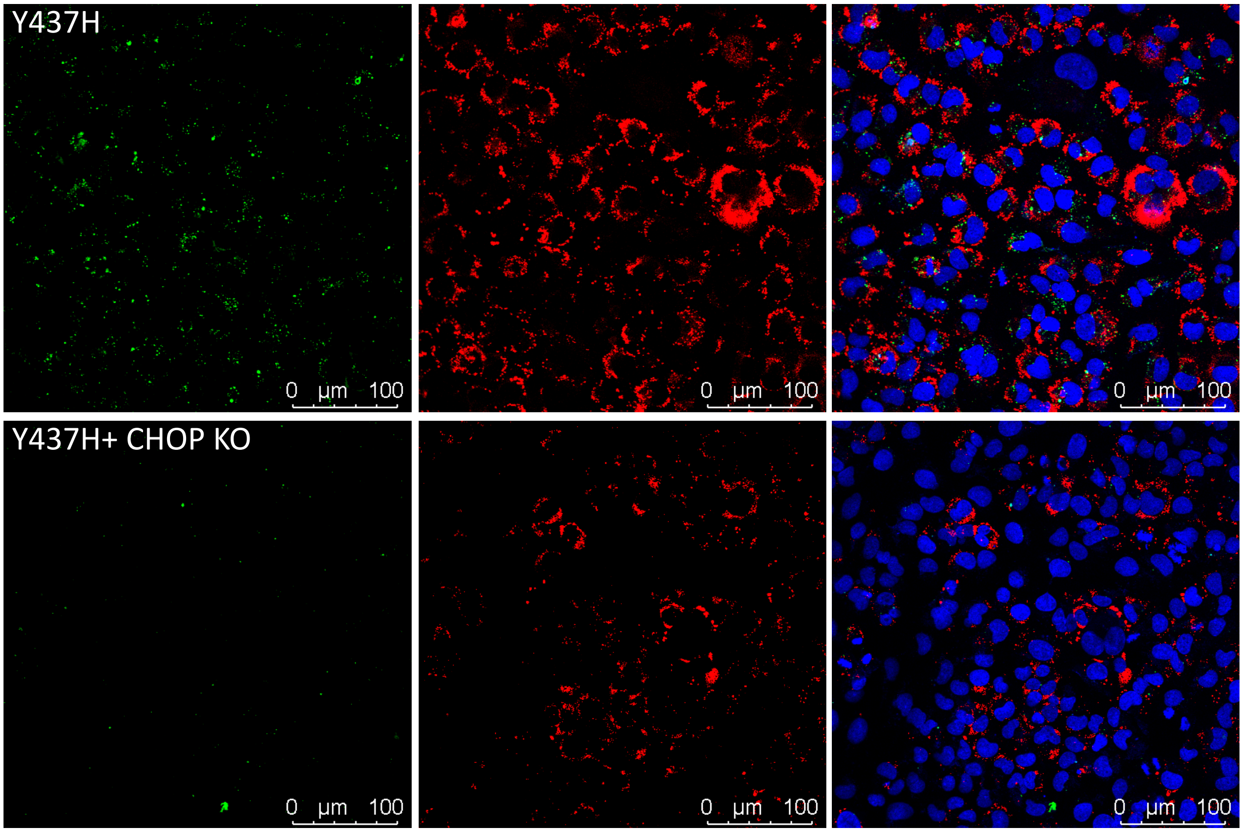

SI.9: Deletion of CHOP reduces mutant myocilin-induced TM cell death. TM3 cells stably expressing DsRed-tagged Y437H mutant of myocilin were analyzed by TUNEL assay. Abundant TUNEL (green) positive cells were observed in GTM3 cells expressing mutant myocilin. Reduction of CHOP dramatically reduced mutant myocilin and also decreased TUNEL positive TM cells. Scale bar is 100µm. N=2 technical replicates.

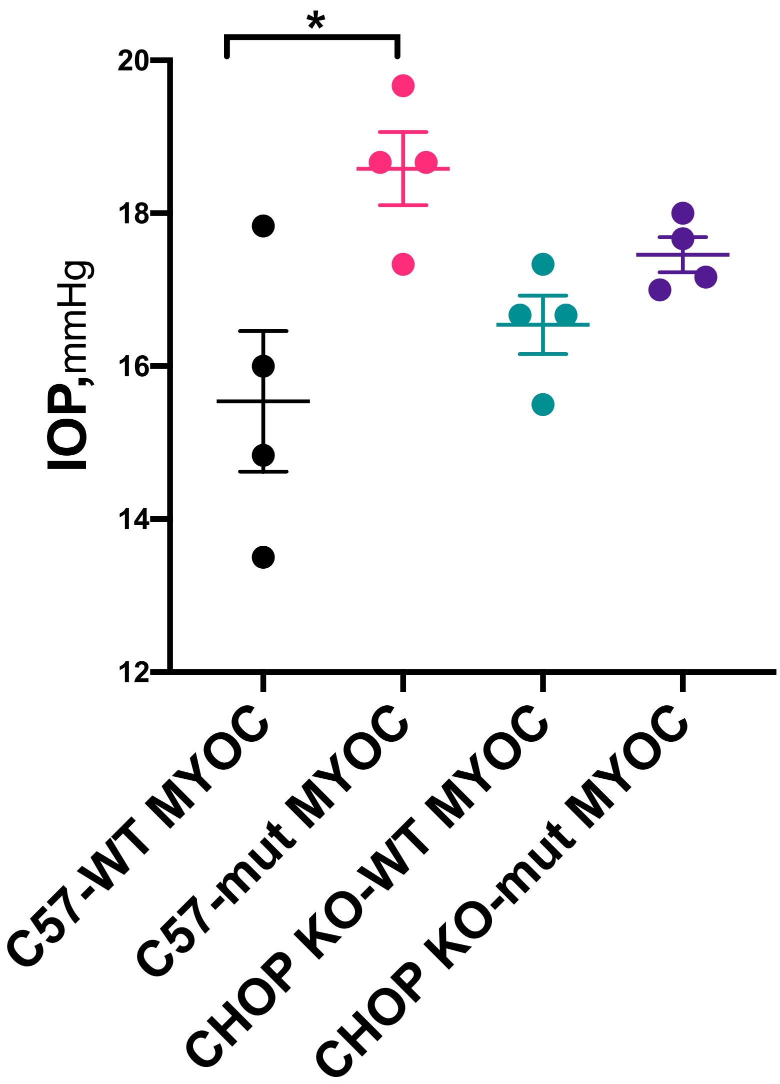

SI.10: *Chop$^{-/-}$* mice prevent mutant myocilin-induced ocular hypertension. Age-matched C57BL/6J and *Chop$^{-/-}$* mice (4-month old) on C57BL/6J background received Ad5 expressing WTMYOC ($1x10^7$ pfu/eye) intravitreal injections in one eye and the contralateral eyes were injected with Ad5 expressing Y437H mutant of MYOC ($1x10^7$ pfu/eye). After 2 weeks of injections, IOPs were recorded. Expression of mutant myocilin significantly elevated IOP in C57BL/6J mice but not in *Chop$^{-/-}$* mice compared to the contralateral eyes injected with WT MYOC. Data are presented as mean ± SEM, N=4 in each group, *p≤0.05, One-way ANOVA.

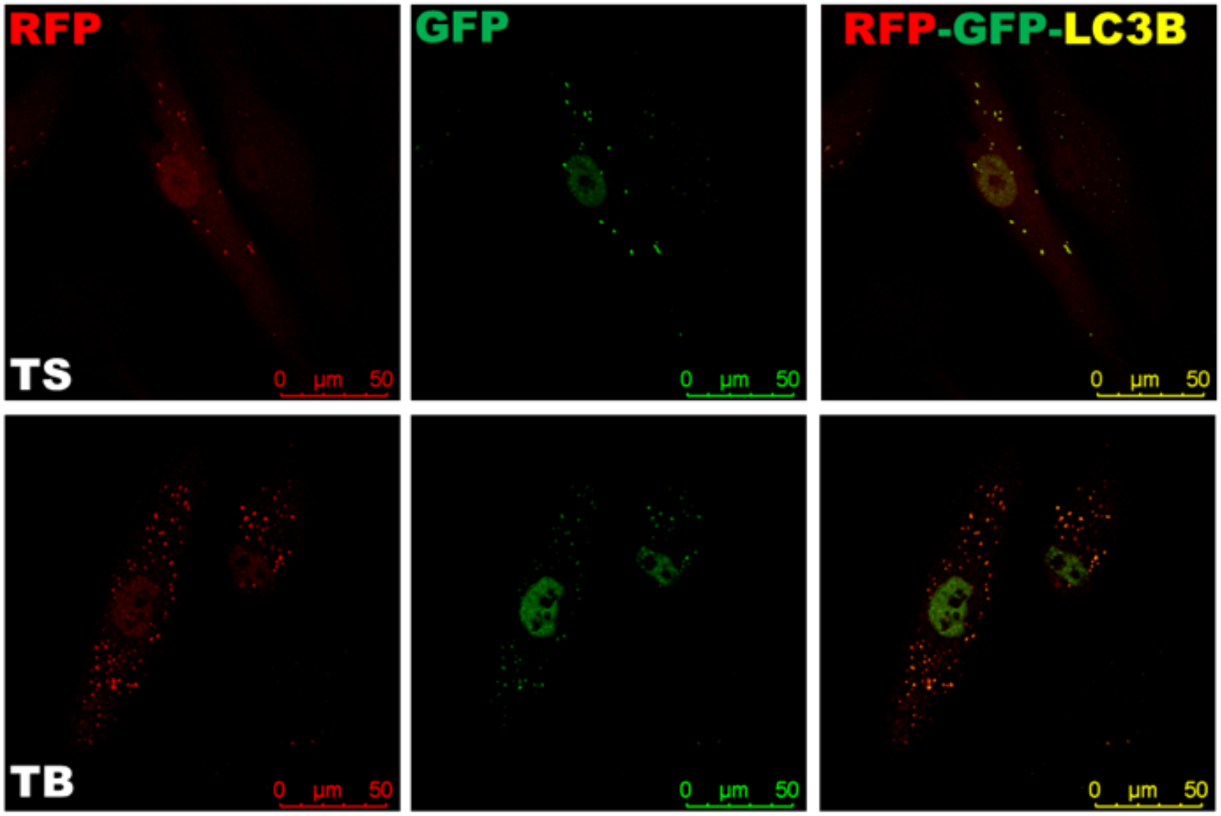

SI.11: Tat-beclin 1 peptide corrects mutant myocilin induced autophagic flux in primary human TM cells. Human primary TM cells (n=3 cell strains) were transduced with Ad5 expressing mutant myocilin for 48 hrs and again transduced with Ad5.mRFP-GFP-LC3 for 24hrs. Cells were further treated with either TS or TB1 peptide for 3hrs. Autophagosomes (yellow punta) and autolysosomes (RFP punta) were counted using a confocal microscope (Leica SP8) and represented graphically in Figure 6E.

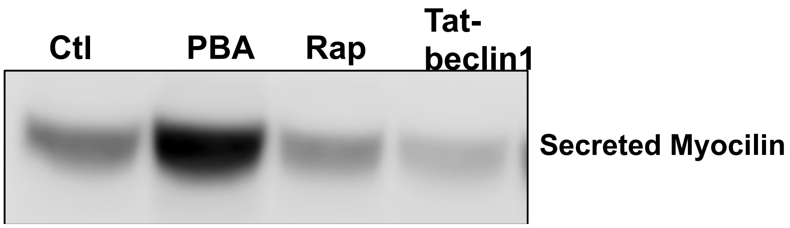

SI.12: Tat-beclin 1 peptide does not increase myocilin secretion. TM3 cells stably expressing mutant myocilin were treated with PBA (5mM) or Rapamycin (100nM) for 12hrs or with Tat-beclin 1 peptide (25μM) for 3 hrs per day for 2 days. The conditioned medium was analyzed for myocilin secretion by Western blot analysis. PBA was used as a positive control. PBA treatment increased mutant myocilin secretion in the medium. However, treatment with autophagy inducers did not increase myocilin secretion in the medium. N=2 replicates.

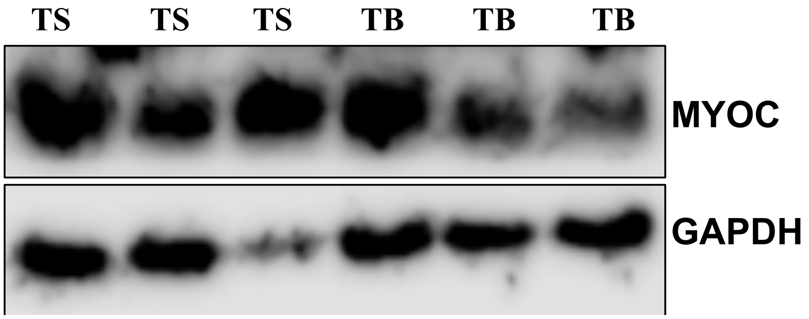

SI.13: Tat-beclin 1 peptide reduces mutant myocilin in anterior segment tissues of *Tg-MYOC*^{Y437H} mice. Western blot analysis of anterior segment tissue lysates from TS or TB treated mice demonstrated that TB1 peptide treatment significantly reduces intracellular myocilin accumulation in *Tg-MYOC*^{Y437H} mice. N=3.

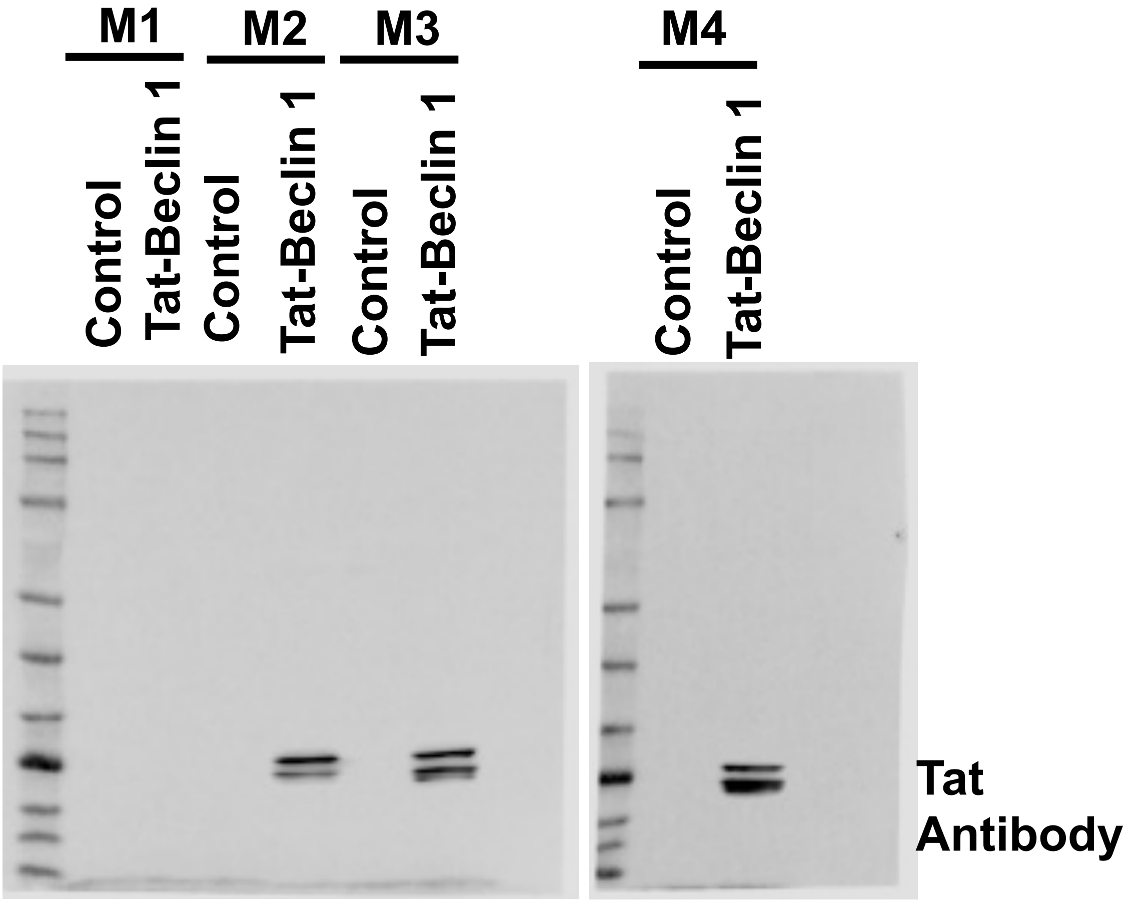

SI.14: Western blot analysis showing the presence of tat-beclin 1 peptide in the aqueous humor of mice treated with topical ocular tat-beclin1 peptide. C57BL/6J mice (n=4) were given topical ocular eye drops of PBS (control) in one eye while the contralateral eyes were treated with tat-beclin1 peptide twice daily for 1 week and aqueous humor (3 µl) was collected. Tat-beclin1 peptide was detected by Western blotting using antibody specific to tat protein. Out of 4 mice, tat-beclin1 peptide was detected in 3 mice. These data indicate that topical ocular delivery of tat-beclin1 peptide penetrates ocular tissues and reaches the TM.

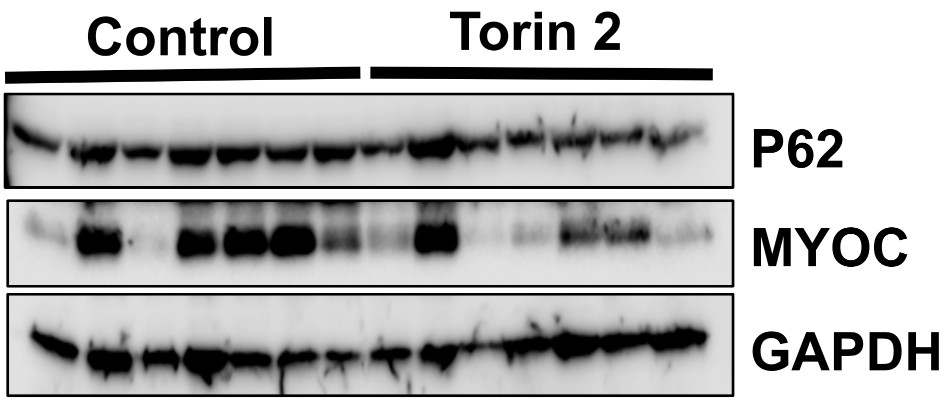

SI.15: Torin 2 reduces mutant myocilin in anterior segment tissues of *Tg-MYOC^{Y437H}* mice. Western blot analysis of anterior segment tissue lysates from vehicle or torin 2 treated mice demonstrated that torin 2 reduces mutant myocilin and p62. N=7 each.

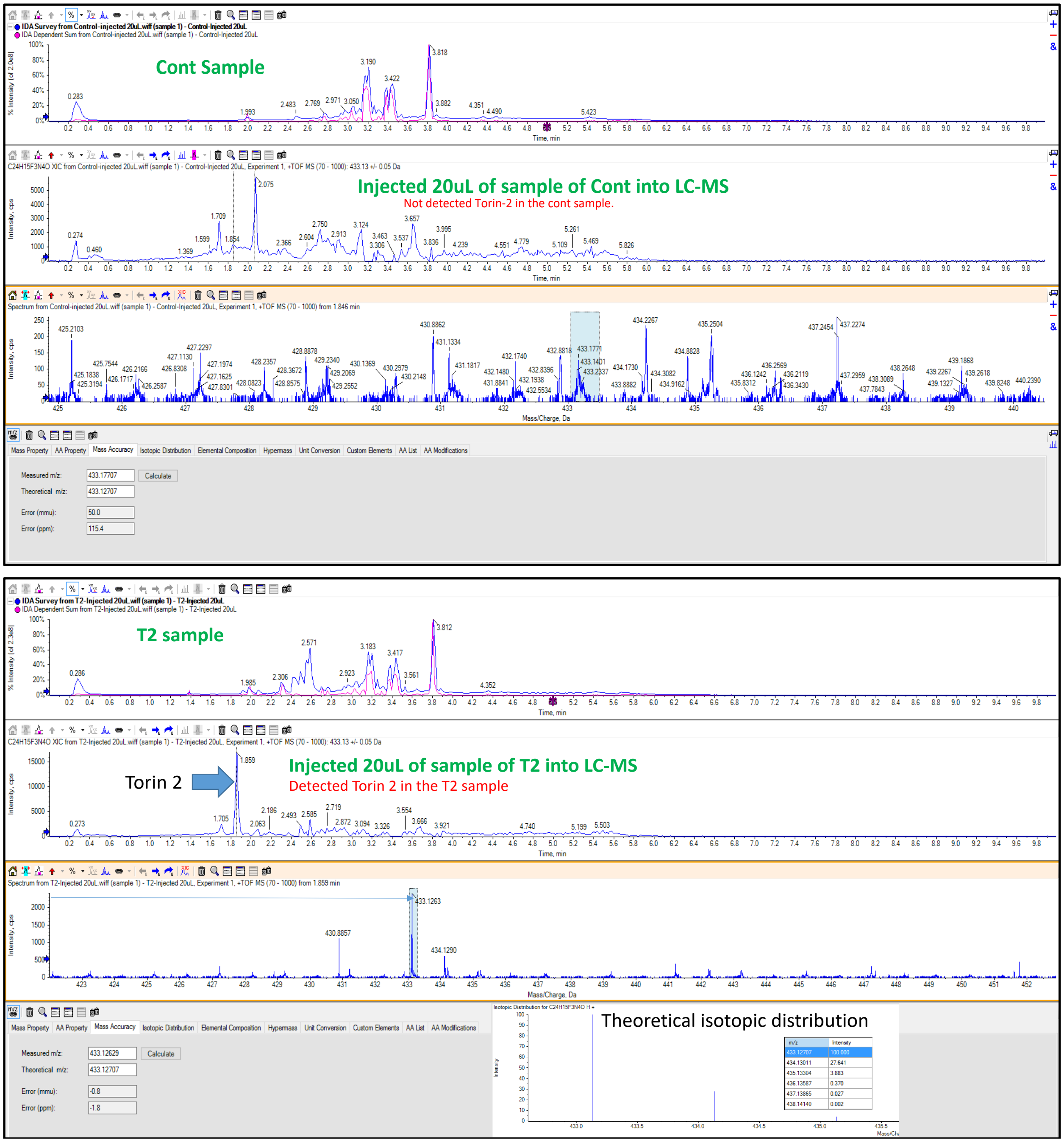

SI.16: Bioavailability of torin 2 in the aqueous humor of mice-treated with topical ocular torin 2. Five WT mice were given topical ocular torin 2 (100 µM) eye drops in one eye while the contralateral eyes received PBS twice daily for a week. Aqueous humor (3-4 µl) was collected from each mouse and samples were pooled together. ~20µl of control and torin 2 treated samples (T2) were sent to UTSW Metabolomics Core facility (https://www.utsouthwestern.edu/research/core-facilities/metabolomics-core.html) along with standard torin 2 compound to perform LC-MS analysis. As shown above, torin 2 was not detected in control sample while it was detected in treated samples indicating bioavailability of torin 2 in ocular tissues.

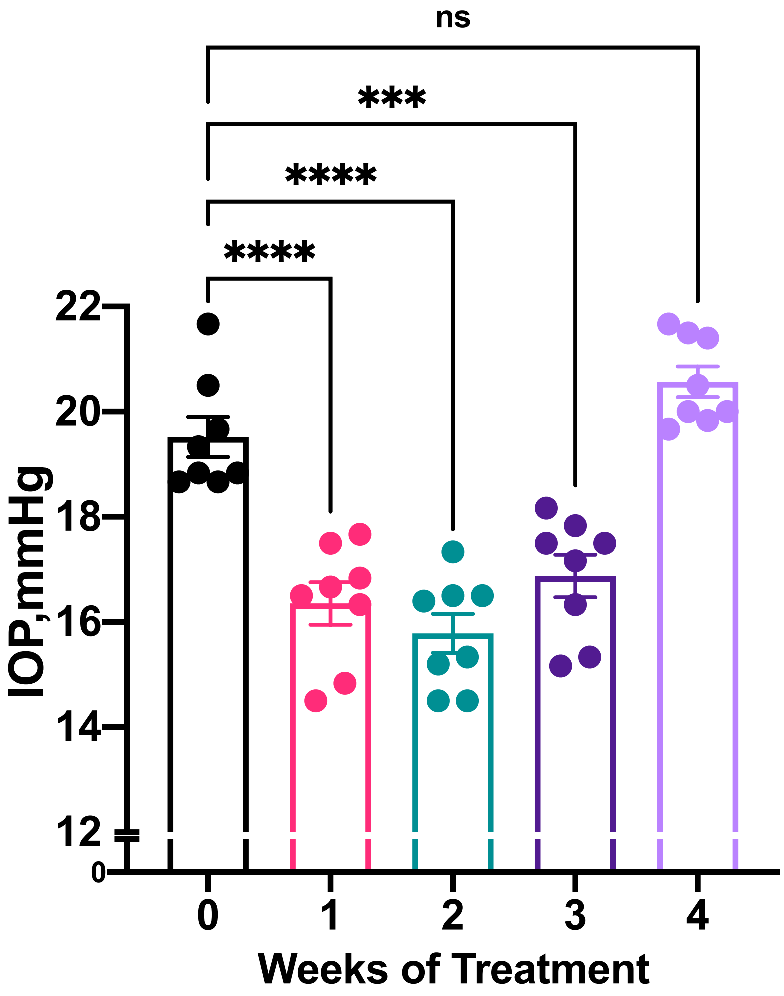

SI.17: Treatment of ocular hypertensive *Tg-MYOC*^{Y437H} mice with torin 2 eye drops reduced IOPs significantly over an extended period of time. IOP was measured in 3 months old WT (n=4) and *Tg-MYOC*^{Y437H} mice (n=8) to ensure IOP elevation in *Tg-MYOC*^{Y437H} mice (0-week). Ocular hypertensive *Tg-MYOC*^{Y437H} mice were then given torin2 eye drops daily for a week (treatment was stopped at the end of the week) and IOPs were monitored for 4 weeks. Starting from 1-week of treatment, torin 2 significantly reduced IOP in *Tg-MYOC*^{Y437H} mice. Interestingly, IOPs remain significantly lower compared to baseline (0-week) at week 2 and 3 despite treatment was stopped. IOPs retuned to baseline level after 4 weeks. These data indicate that torin 2 treatment reduces IOP significantly for an extended period of time. 1-way ANOVA; **** $p<0.0001$, *** $p=0.0001$.

Full unedited blot for figure. 1E

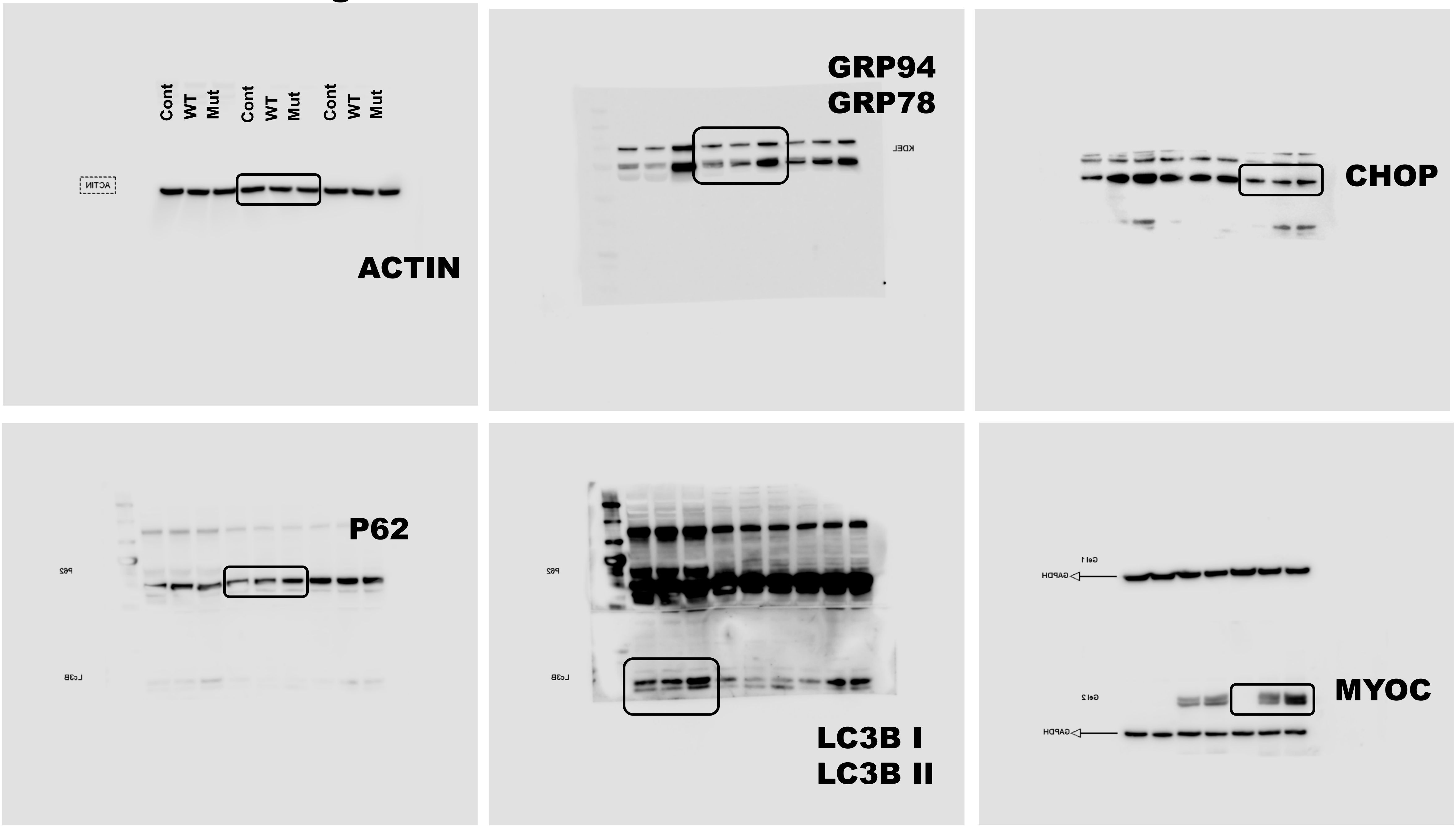

Note: All 3 set of samples (n=3)were utilized for densitometric analysis of Fig.1F. The best representative band for the marker selected randomly from all 3 set of samples

Full unedited blot for figure. 2B

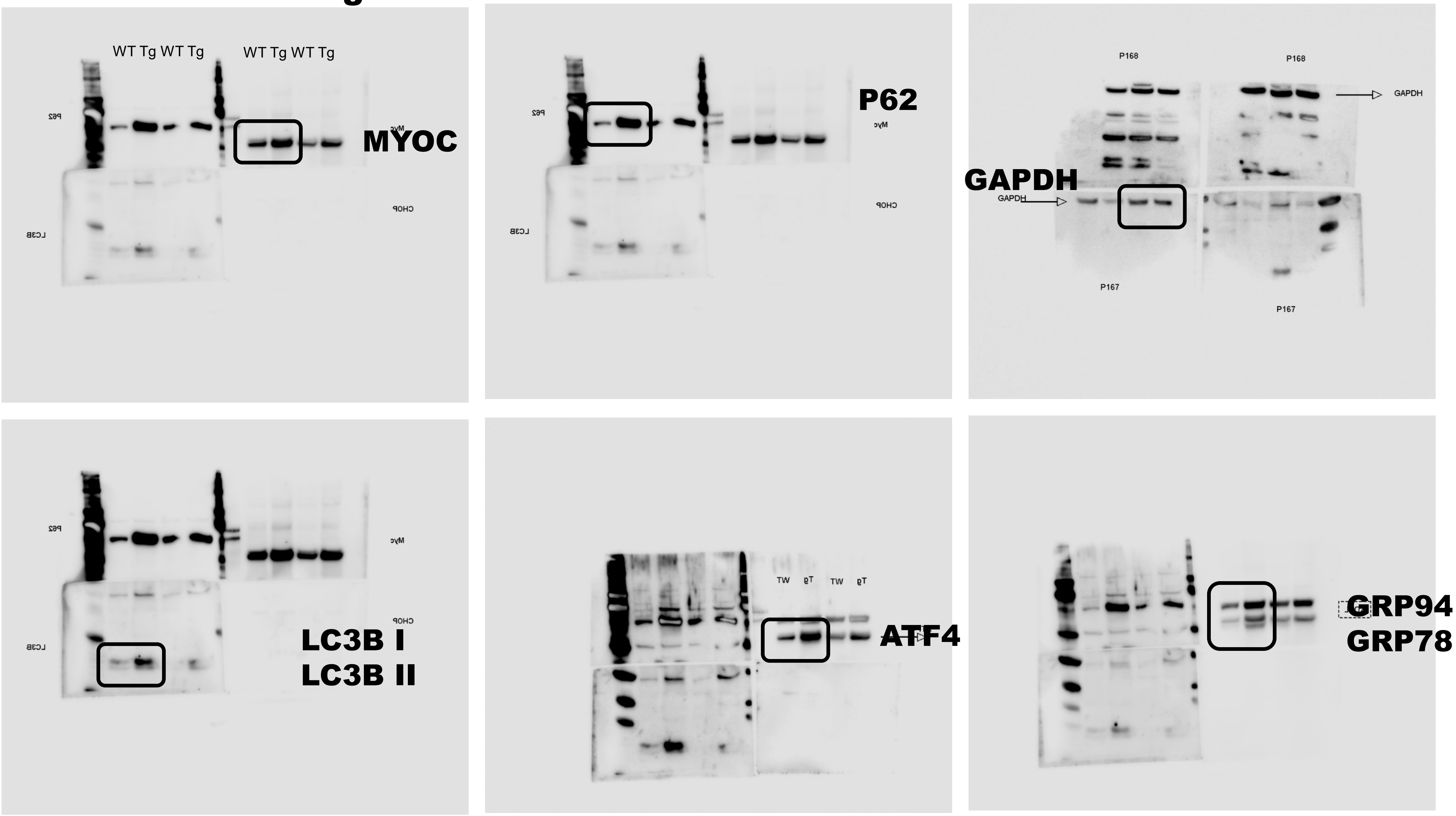

Note: The same samples with the same concentrations loaded twice on same blot to get all the markers mentioned.

Full unedited blot for figure. 2B

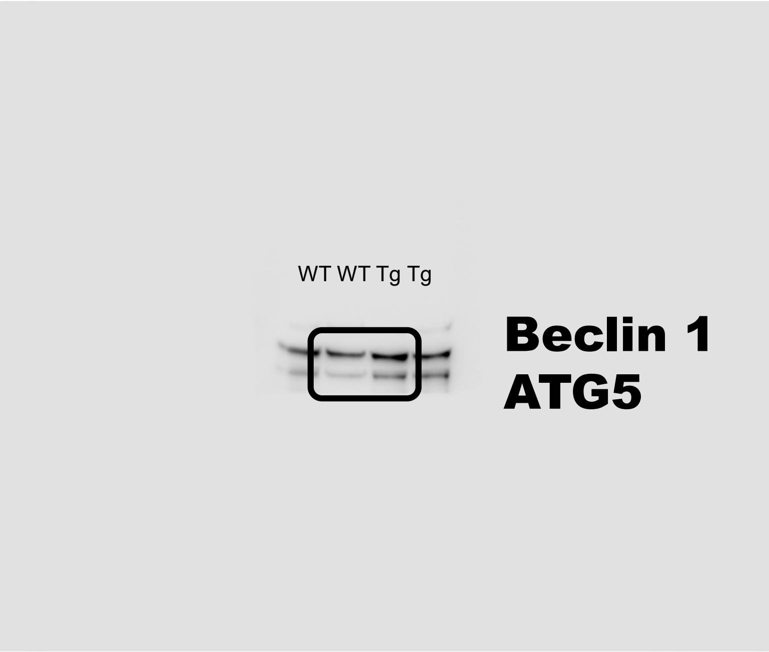

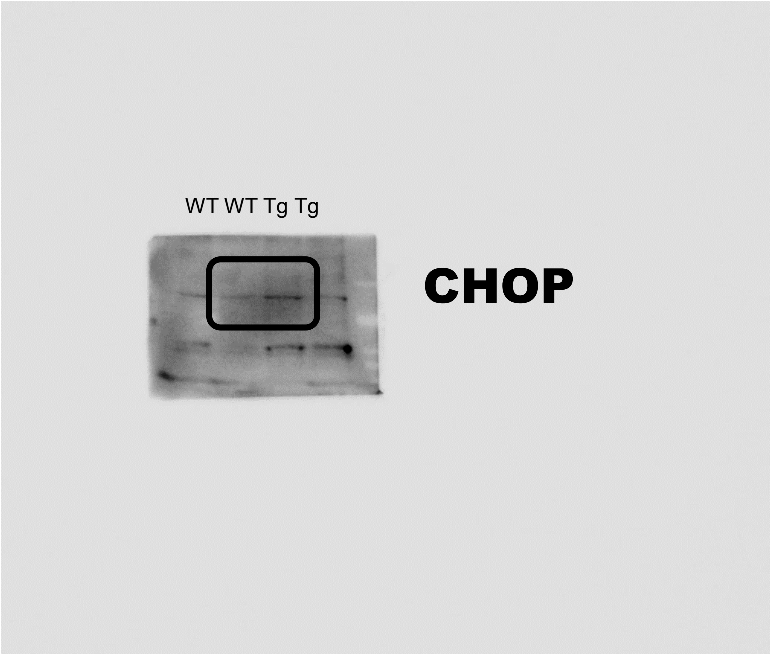

Note: The same samples with the same concentrations loaded on different blots to get all the markers mentioned.

Full unedited blot for figure. 3A

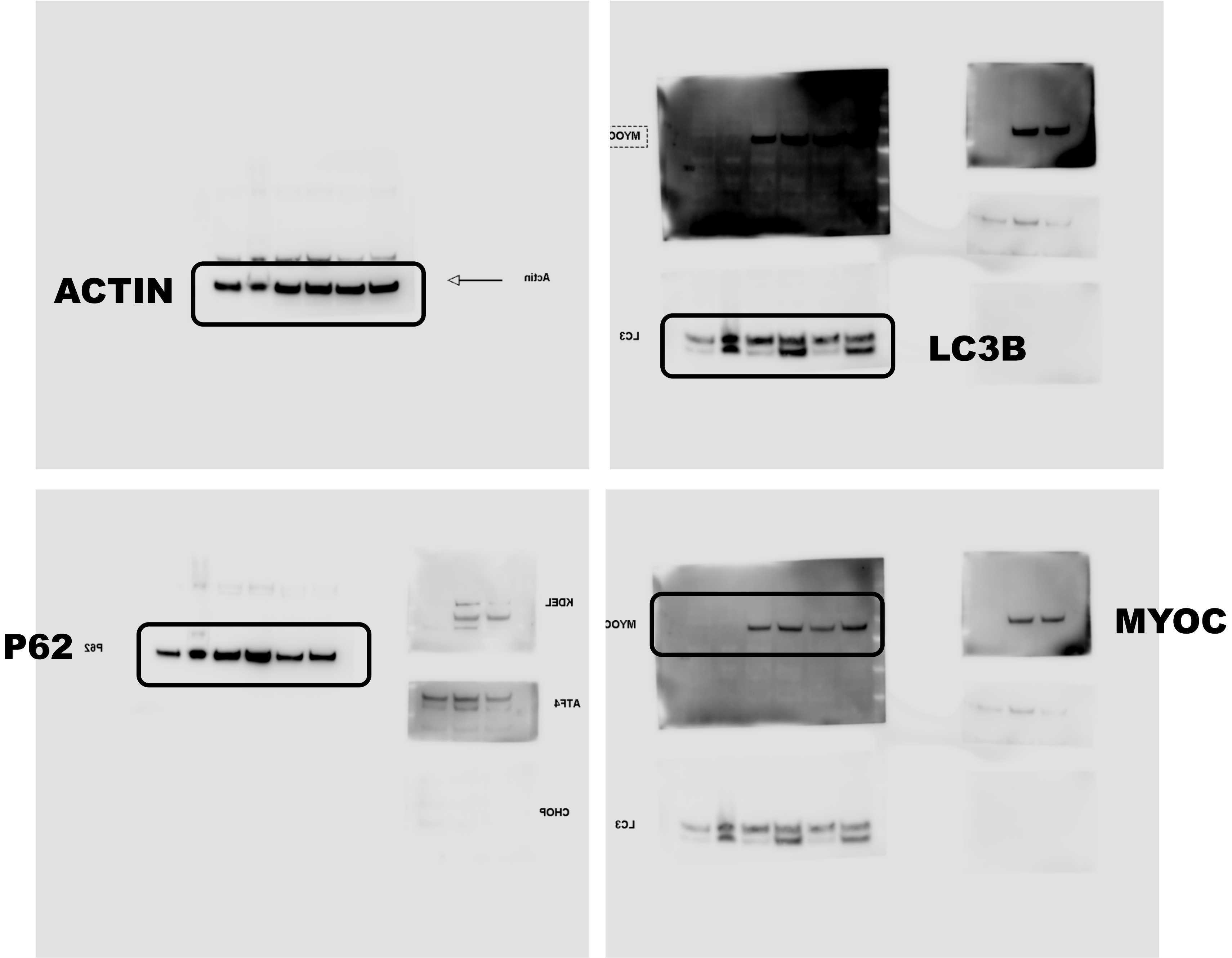

Full unedited blot for figure. 3D

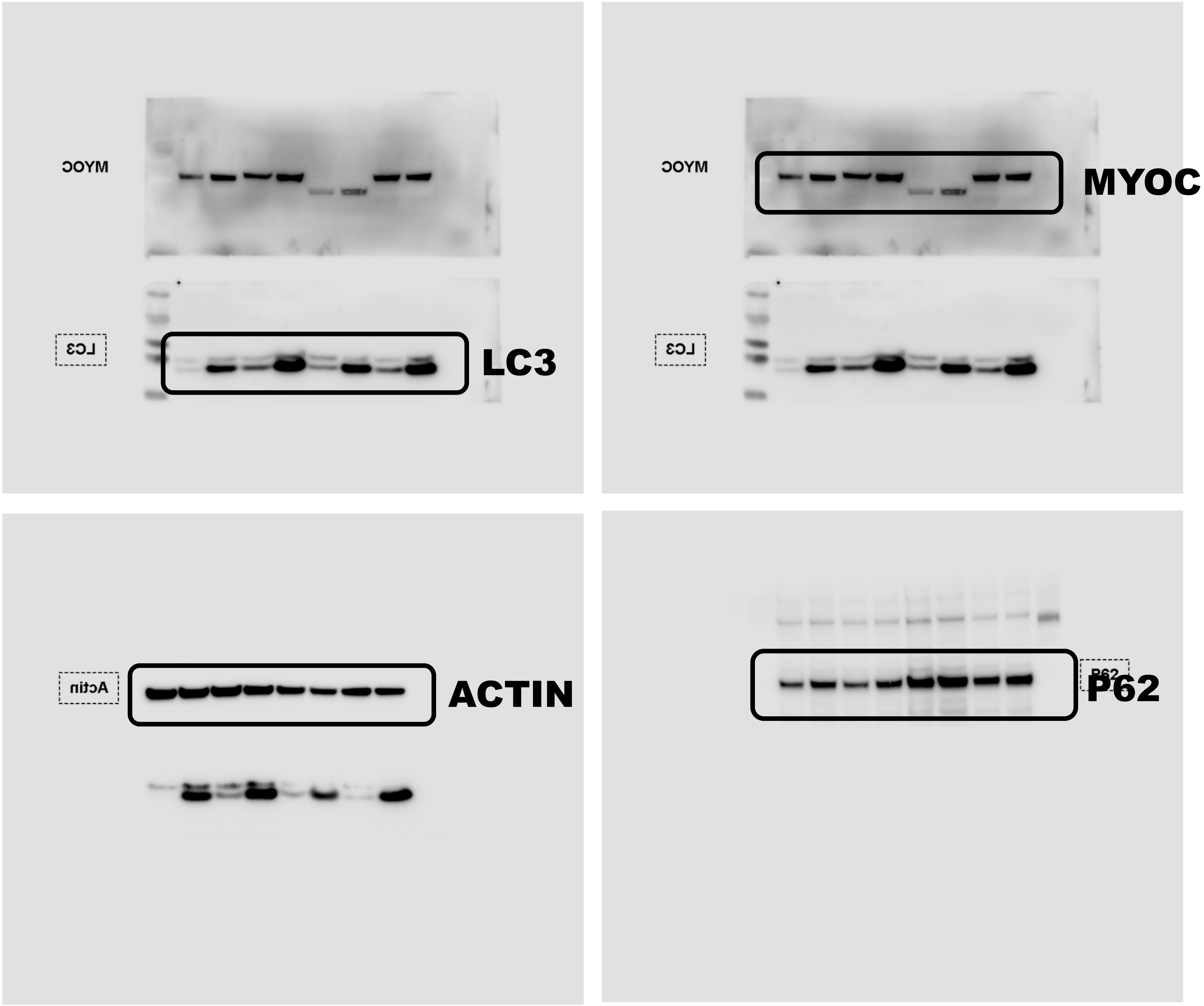

Full unedited blot for figure. 3E

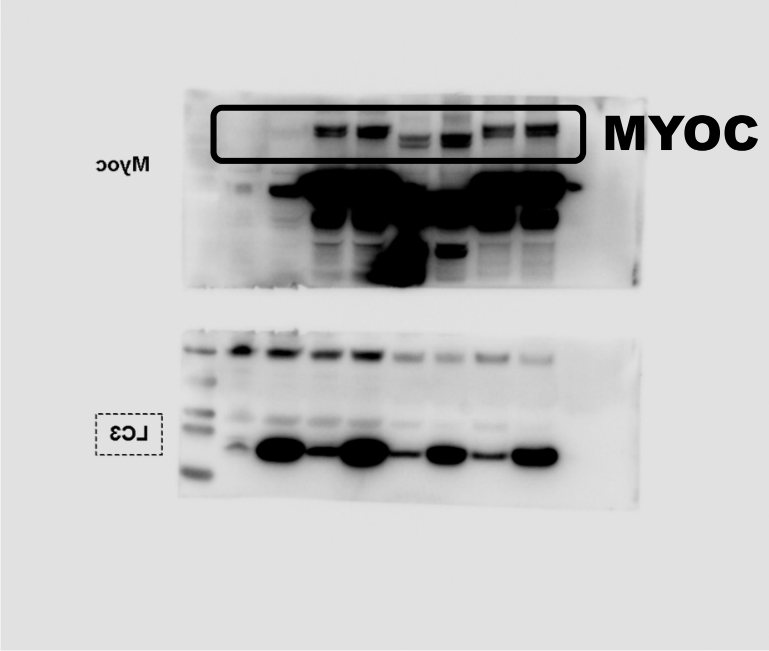

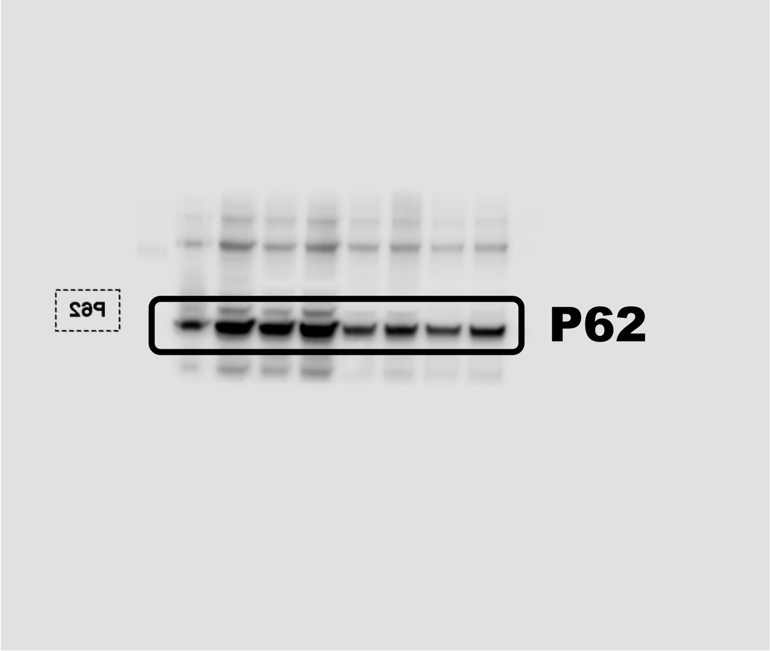

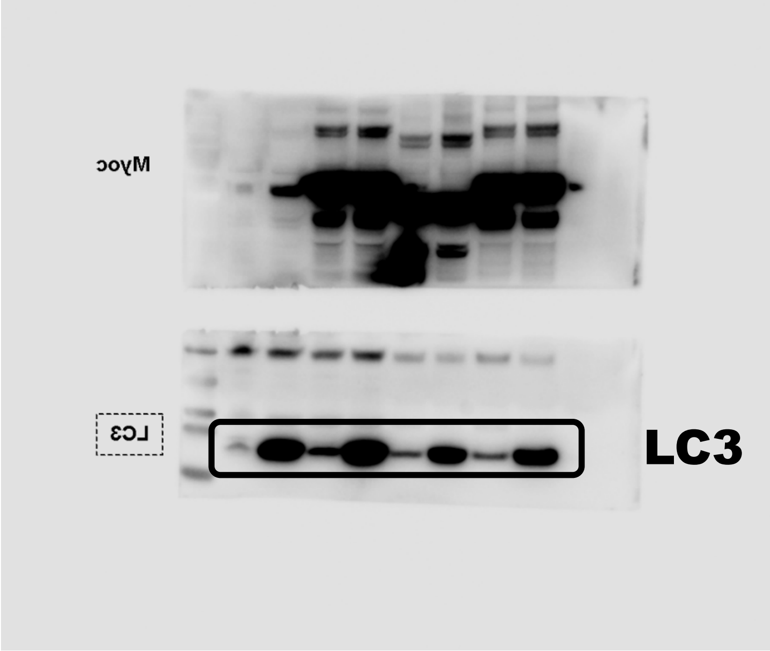

Full unedited blot for figure. 4C

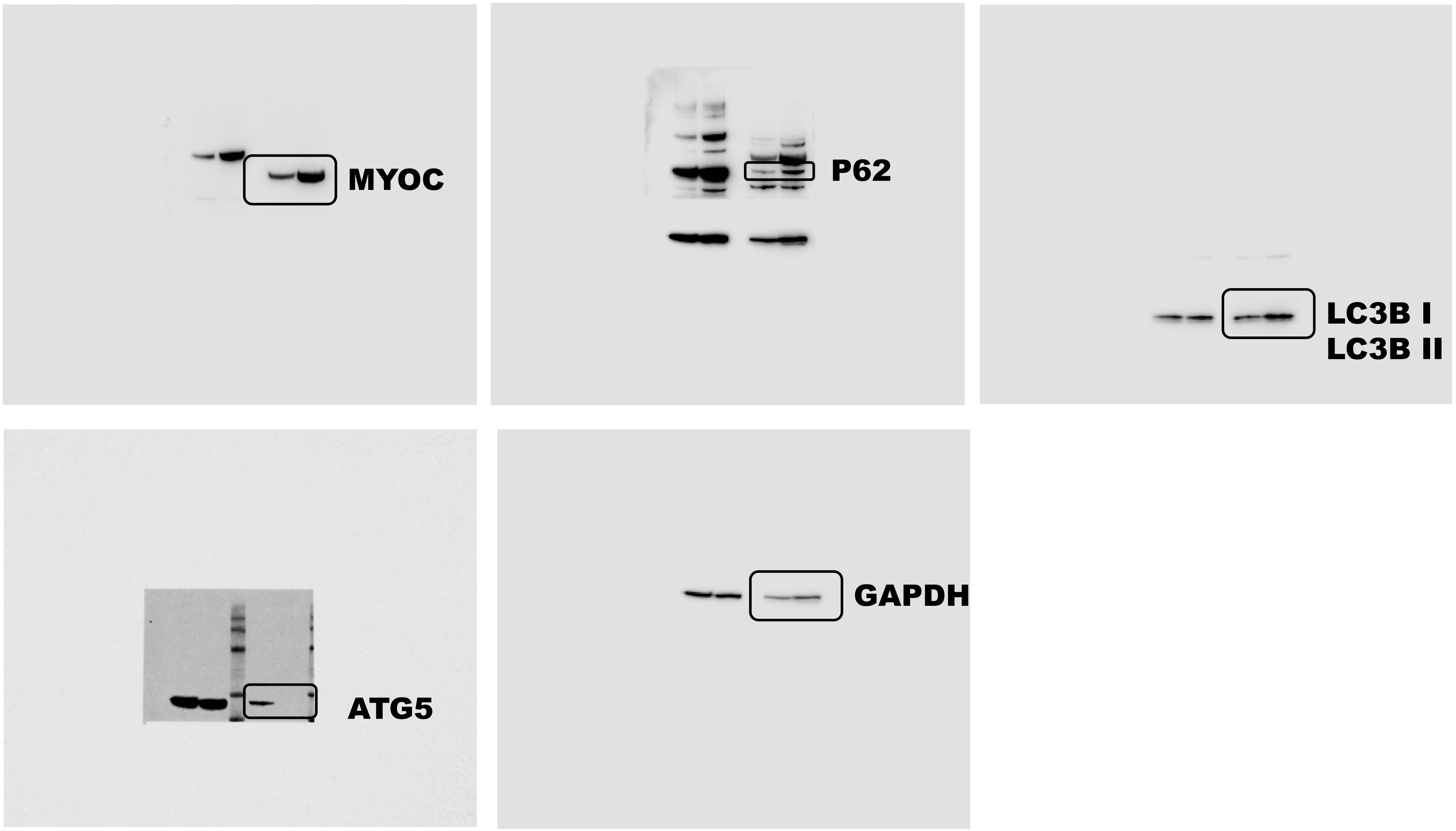

Full unedited blot for figure. 5A

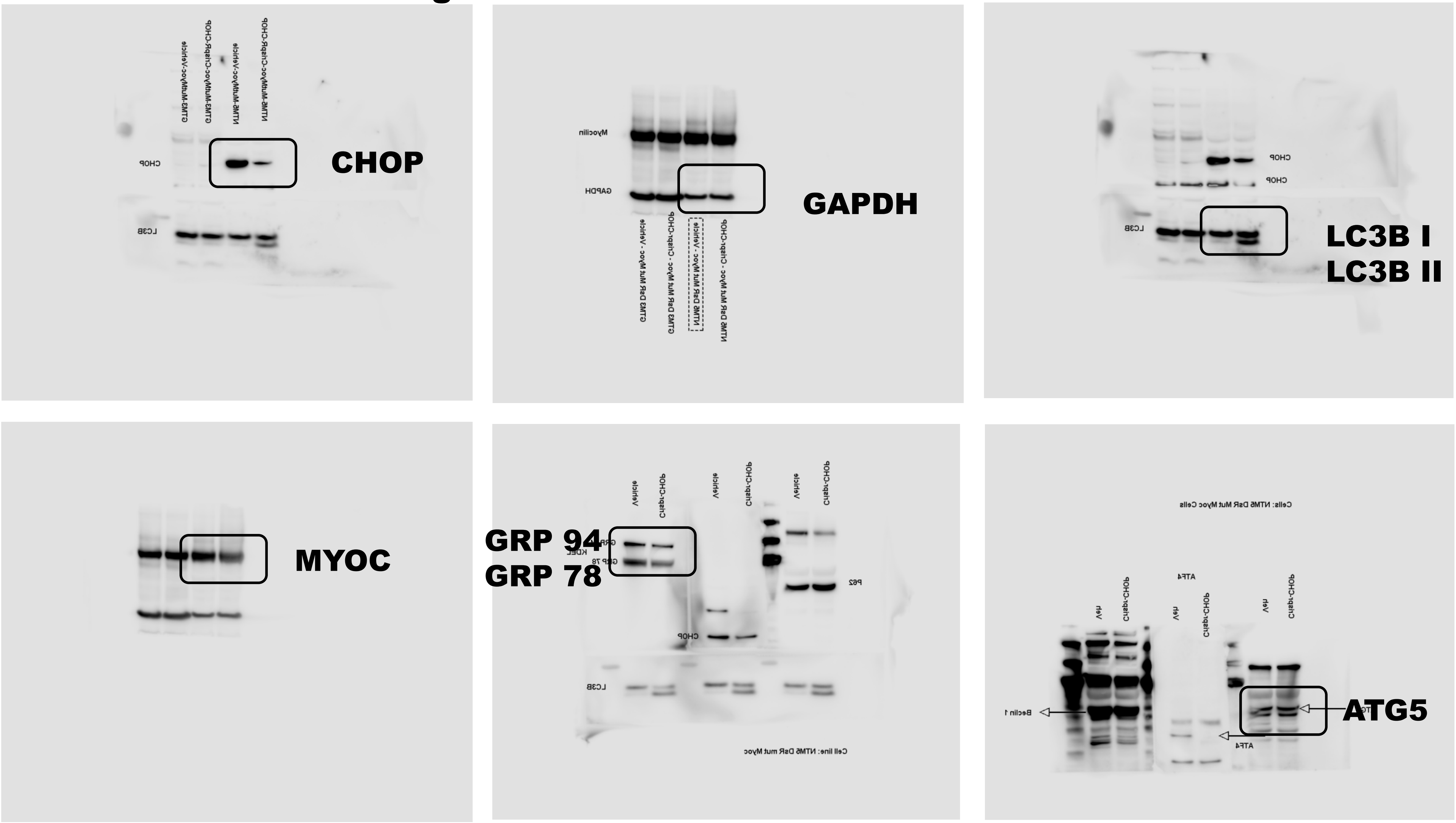

Note: The same samples with the same concentrations loaded twice or thrice on either same or different blots to get all the markers mentioned.

Full unedited blot for figure. 5A

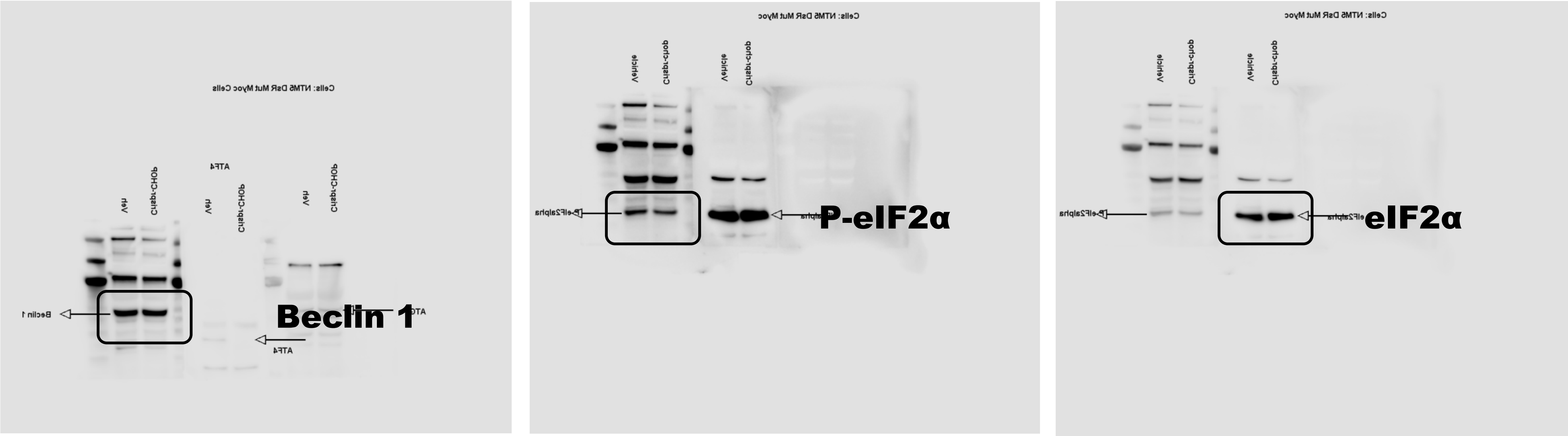

Note: The same samples with the same concentrations loaded twice or thrice on either same or different blots to get all the markers mentioned.

Full unedited blot for figure. 5E

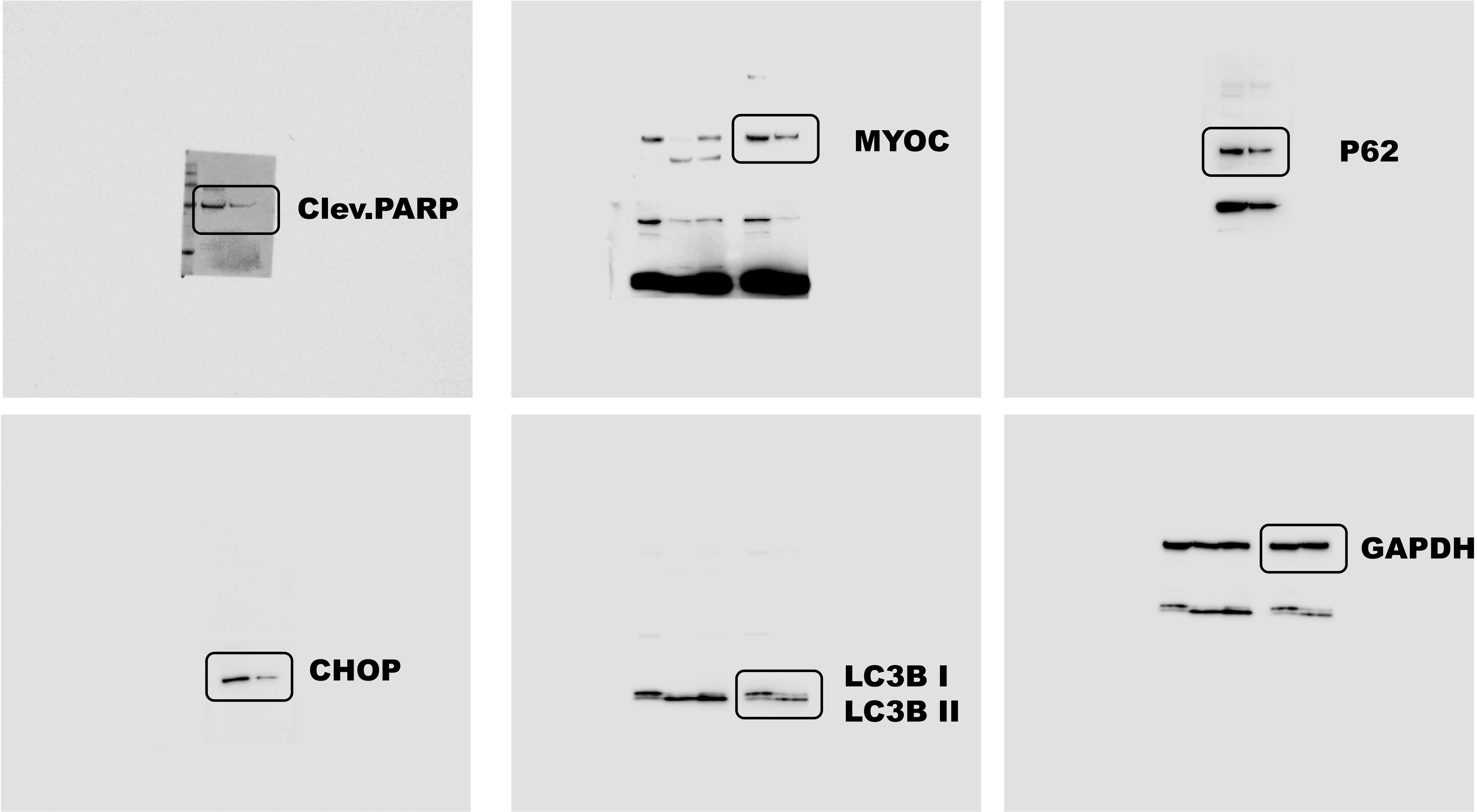

Full unedited blot for figure. 5G

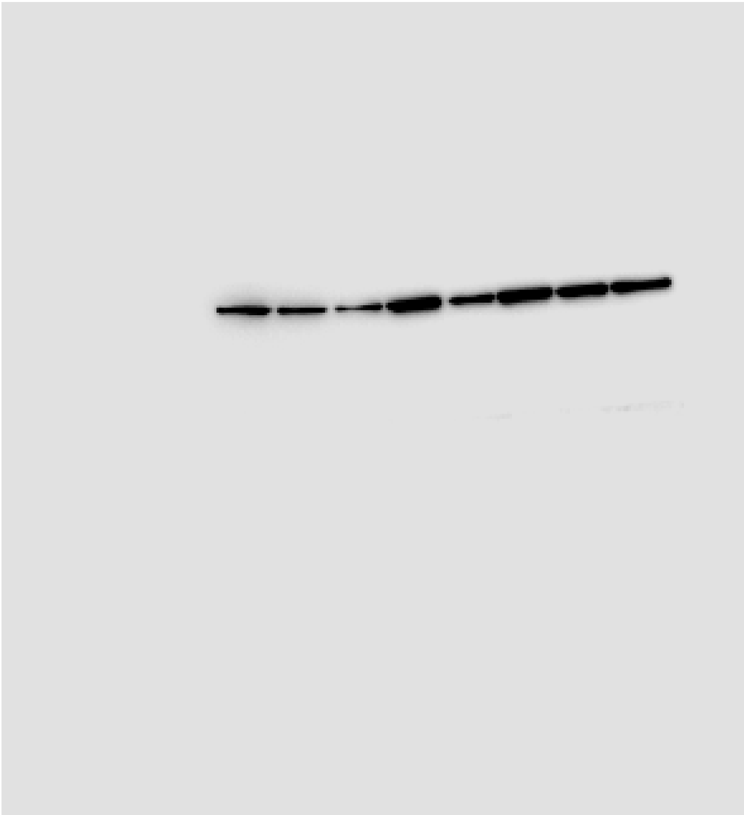

GAPDH

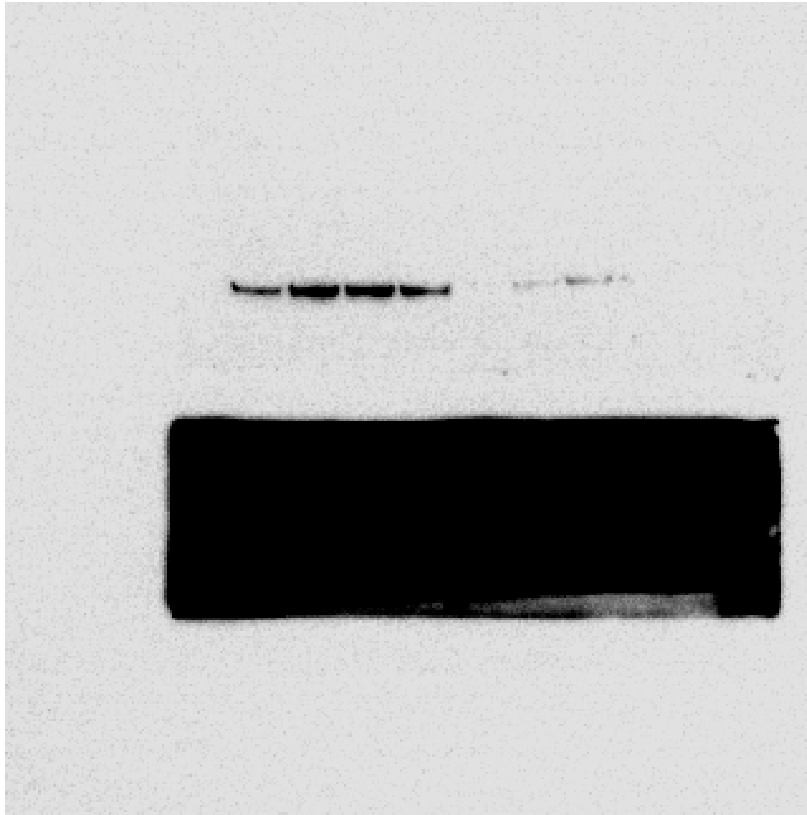

MYOC

Full unedited blot for figure. 6A

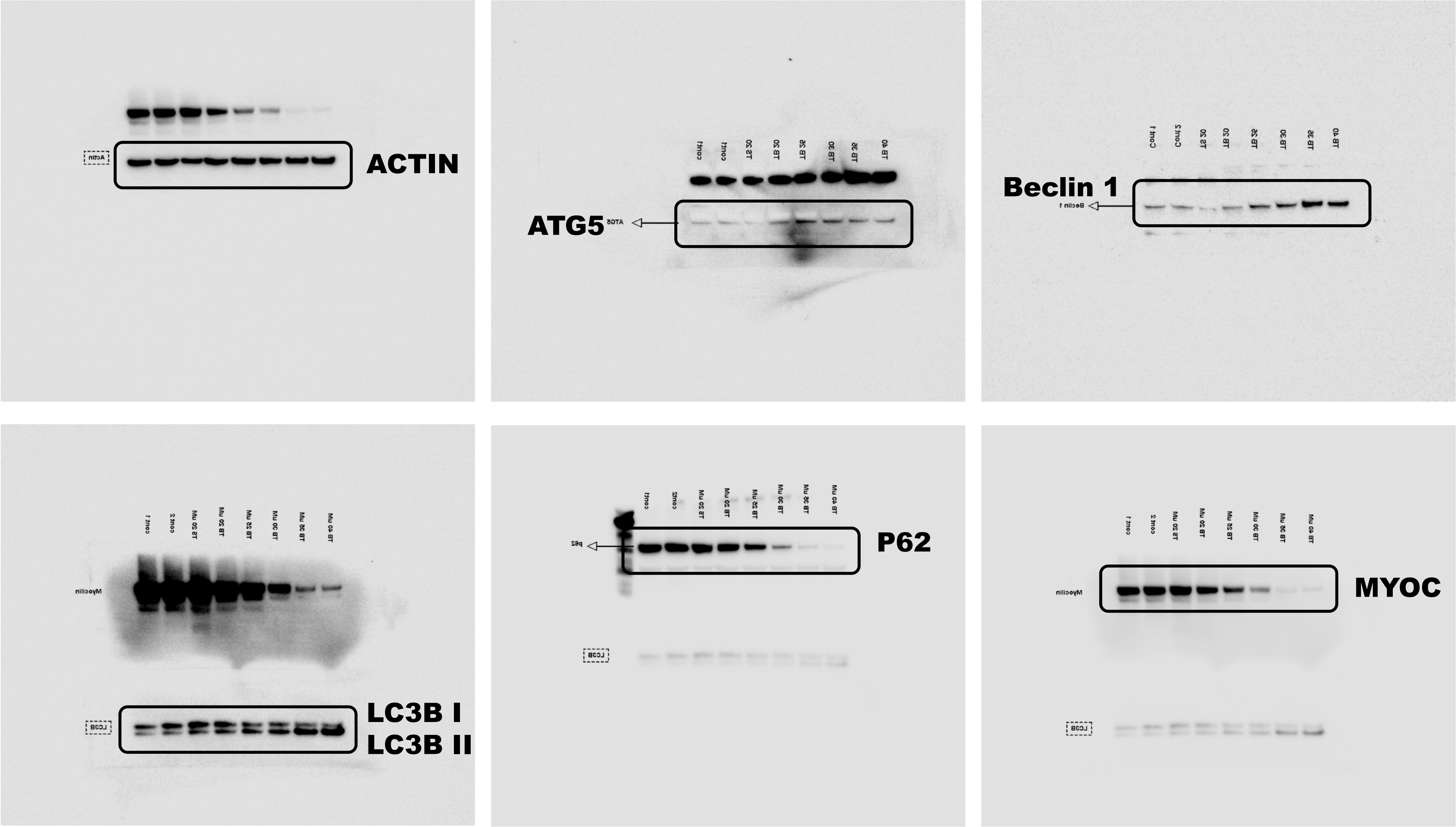

Full unedited blot for figure. 7A

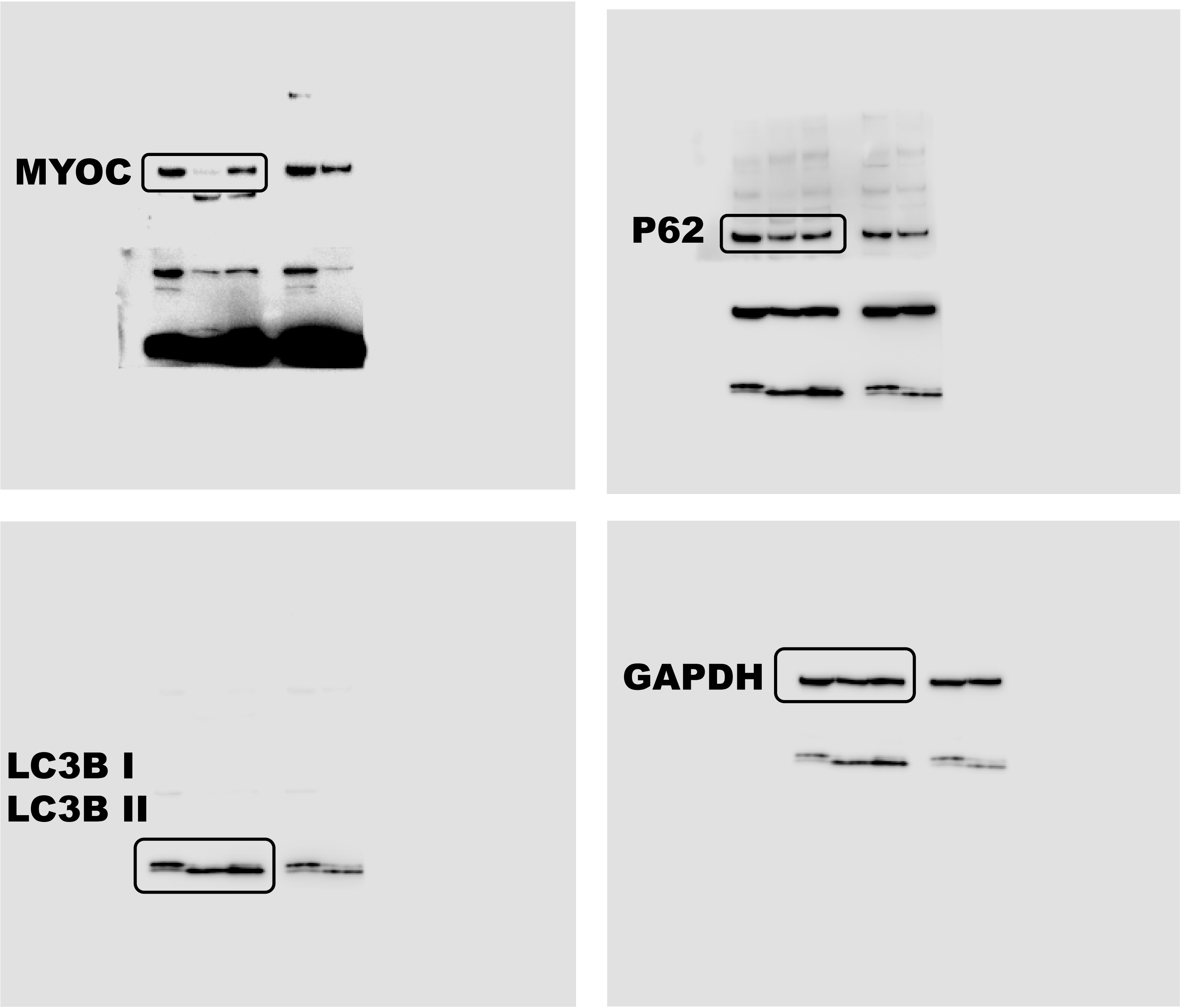

Full unedited blot for supplementary figure. 1B

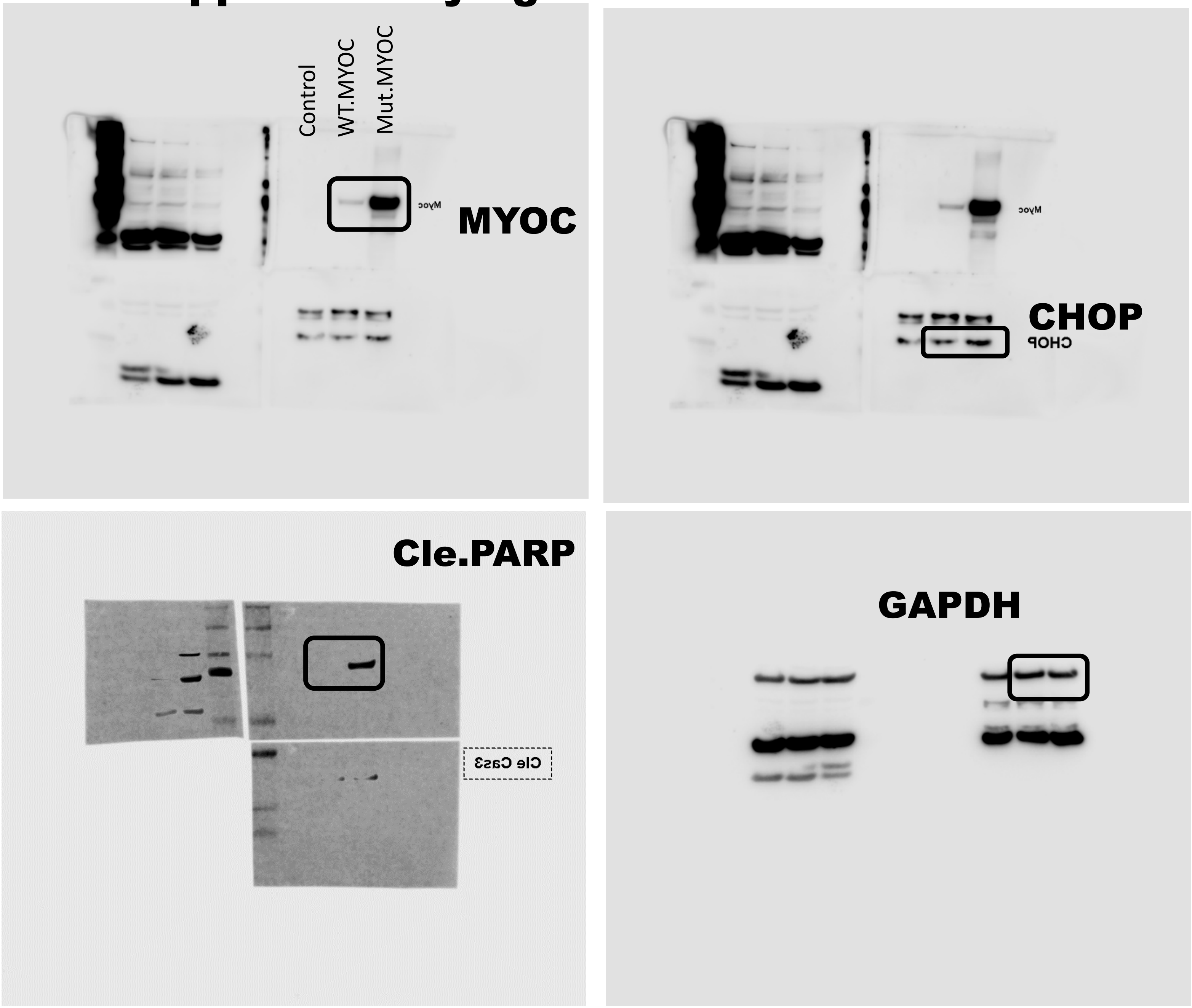

Full unedited blot for supplementary figure. 2A

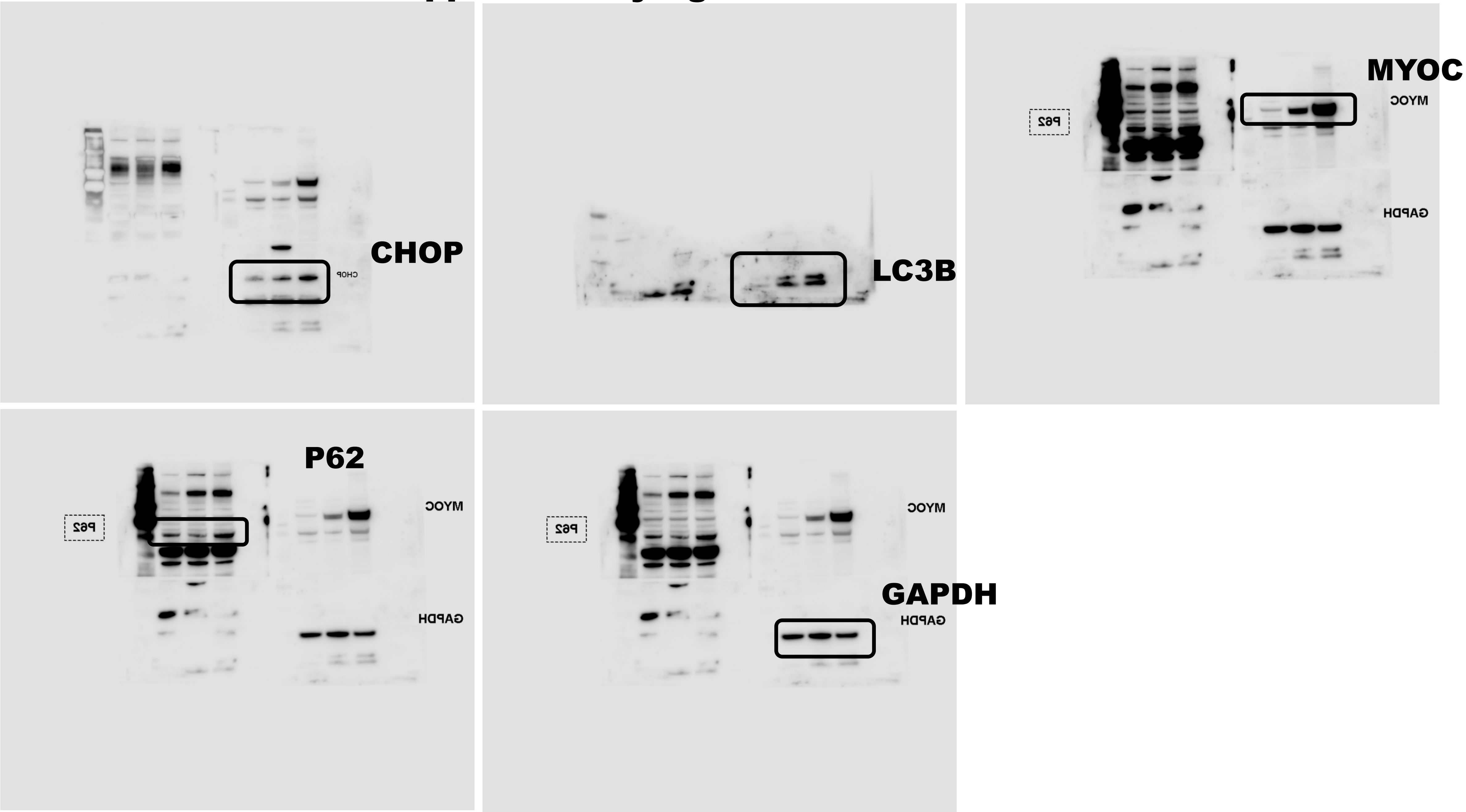

Note: The same samples with the same concentrations loaded twice on same blot to get all the markers mentioned.

Full unedited blot for supplementary figure. 4A

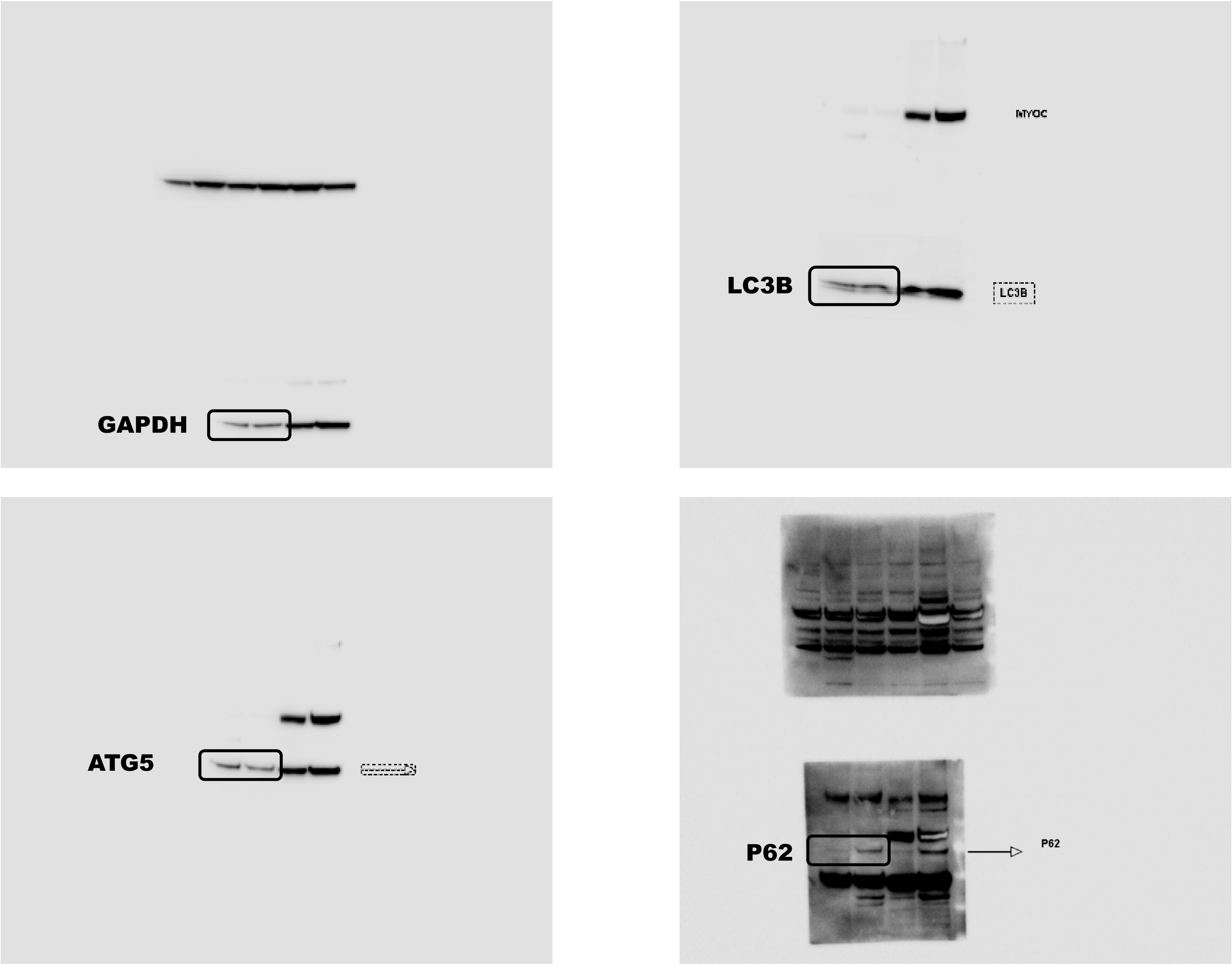

Full unedited blot for supplementary figure. 4B

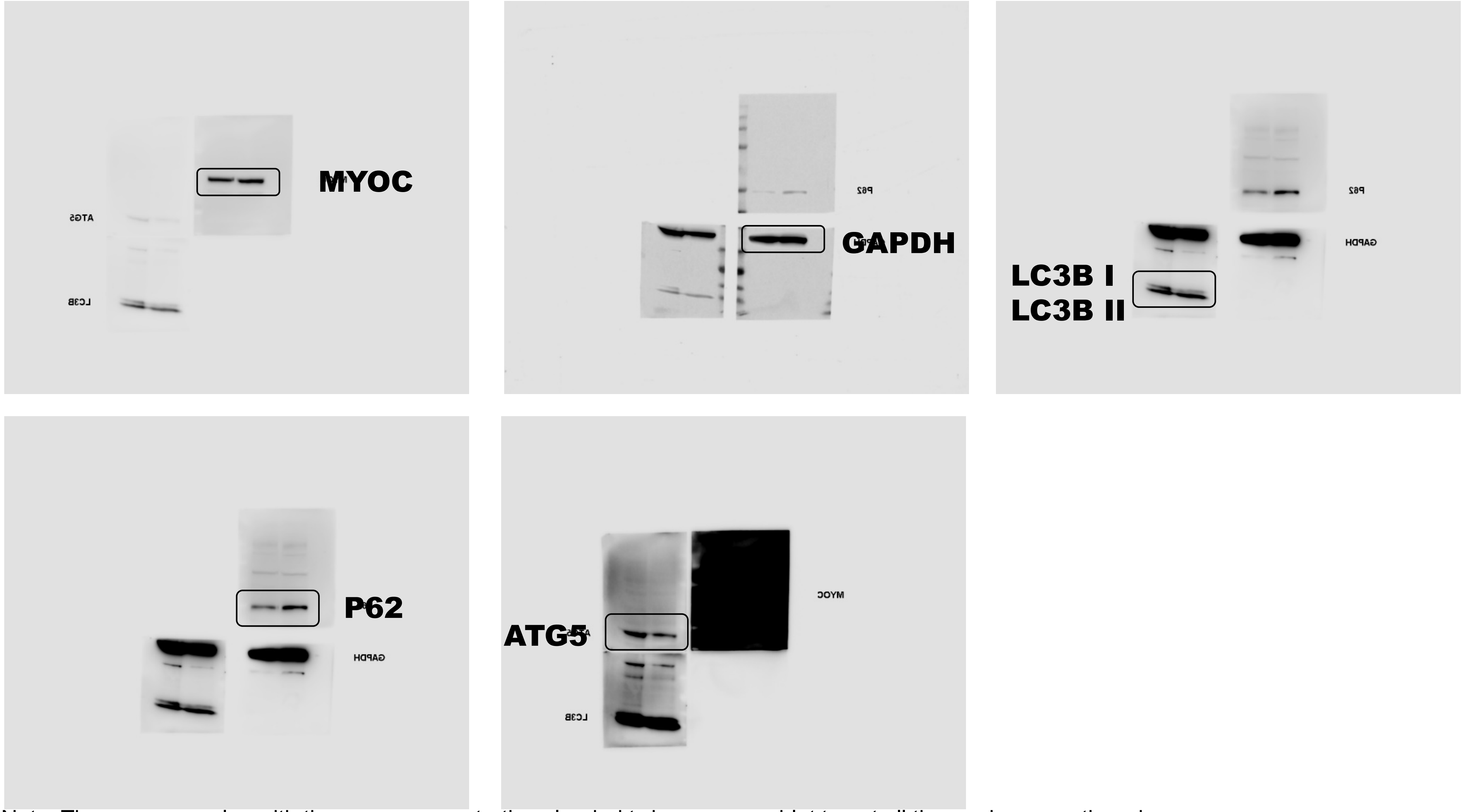

Note: The same samples with the same concentrations loaded twice on same blot to get all the markers mentioned.

Full unedited blot for supplementary figure. 12

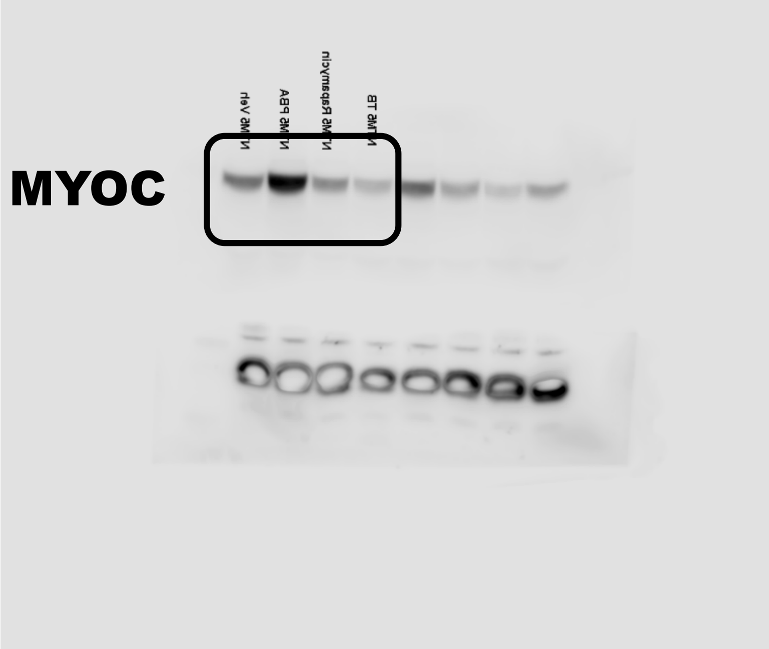

Full unedited blot for supplementary figure. 13

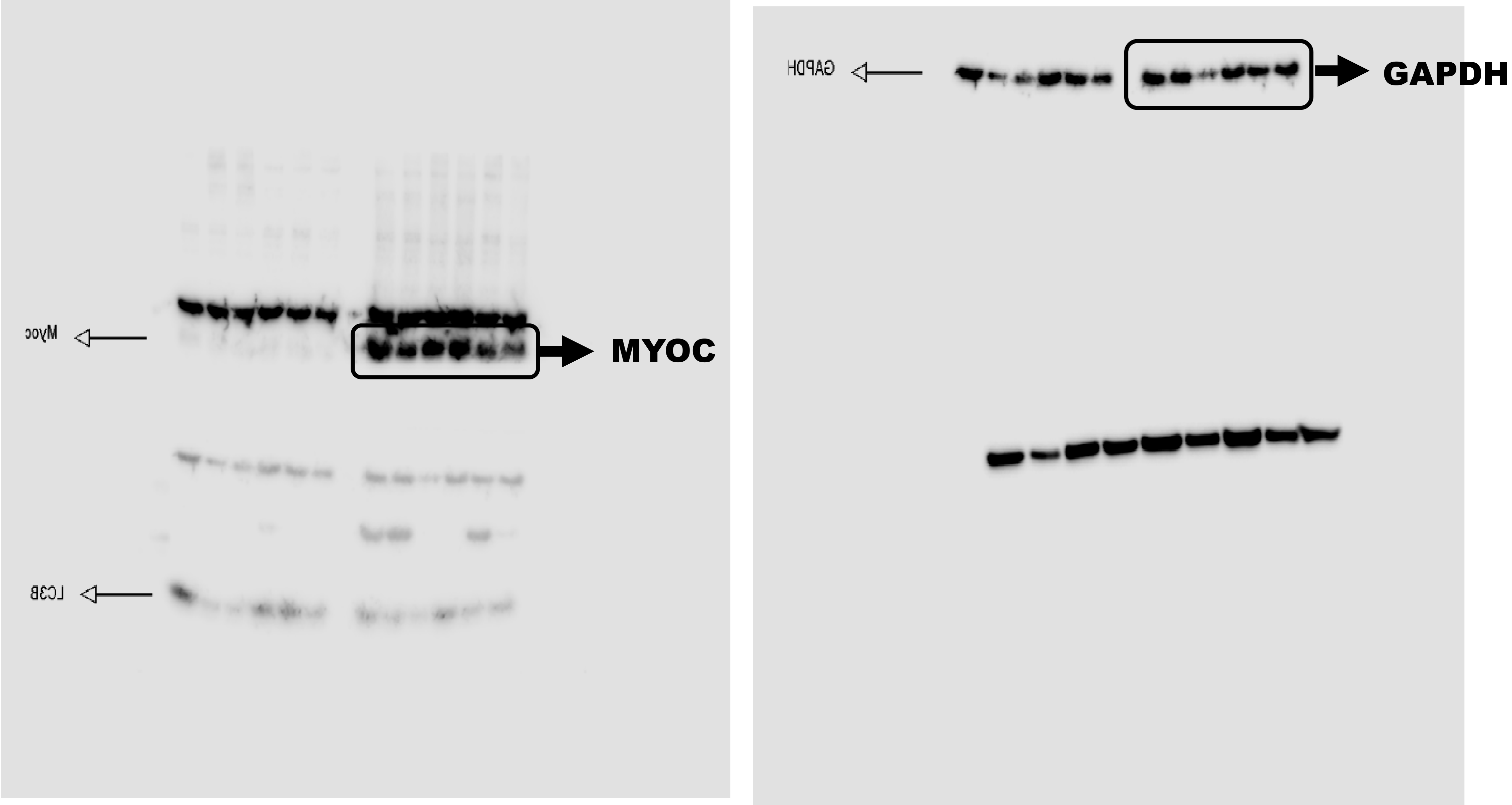

Full unedited blot for supplementary figure. 15

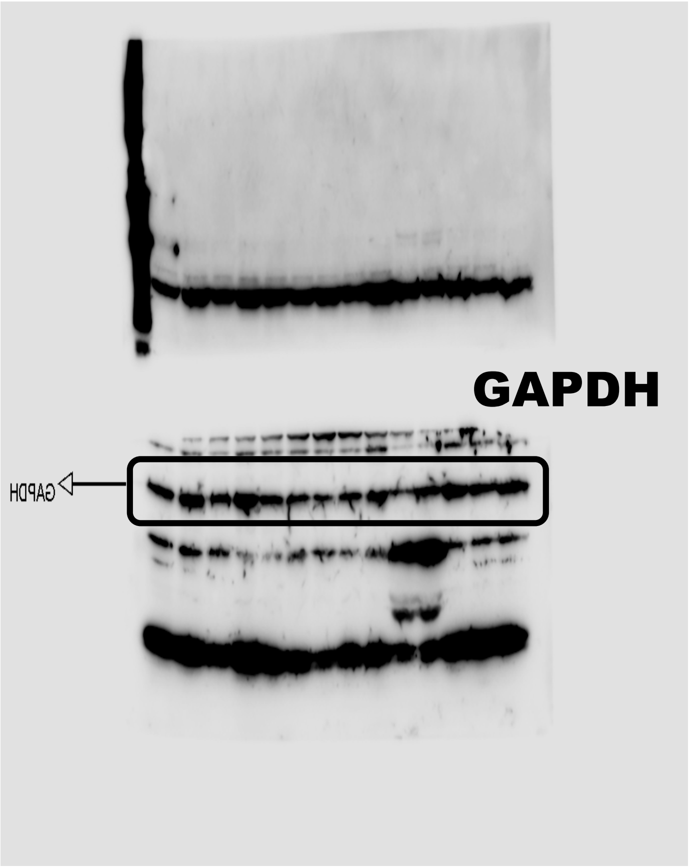

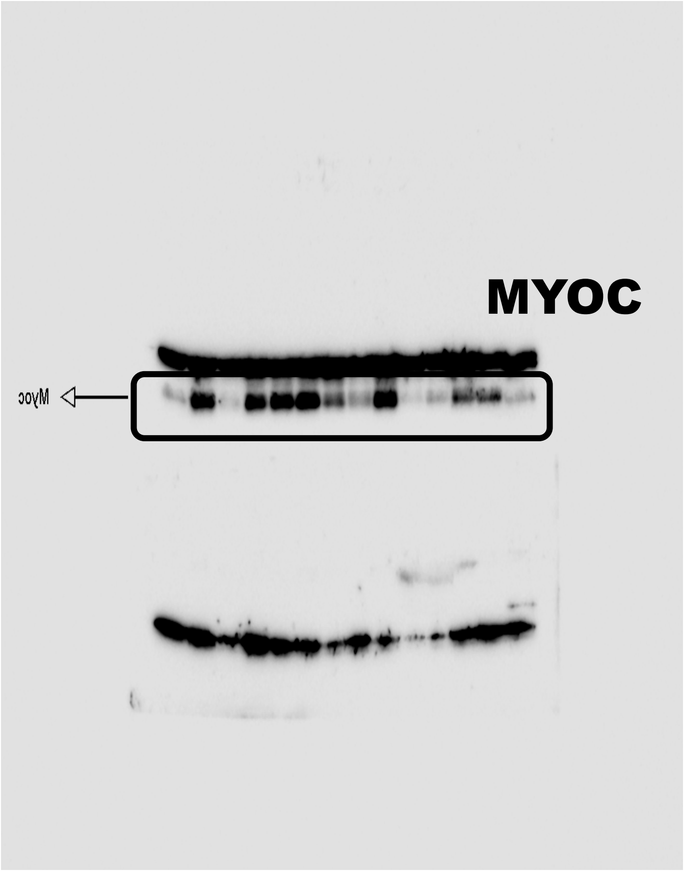

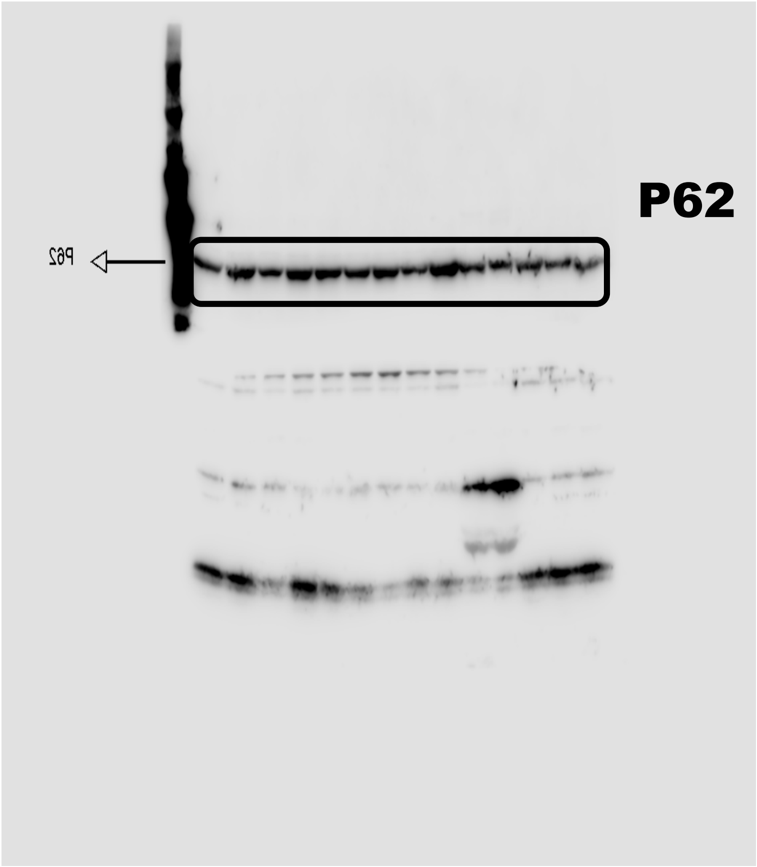

Supplement: Supplemental data [file jciinsight-6-143359-s136.pdf]
